# Post-licensure safety evaluation of dihydroartemisinin piperaquine in the three major ecological zones across Ghana

**DOI:** 10.1371/journal.pone.0174503

**Published:** 2017-03-30

**Authors:** Abraham R. Oduro, Seth Owusu-Agyei, Margaret Gyapong, Isaac Osei, Alex Adjei, Abena Yawson, Edward Sobe, Rita Baiden, Martin Adjuik, Fred Binka

**Affiliations:** 1 Navrongo Health Research Centre, Navrongo, Ghana; 2 School of Public Health, University of Ghana, Legon, Ghana; 3 INDEPTH-Network, Accra, Ghana; 4 Kintampo Health Research Centre, Kintampo, Ghana; 5 Dodowa Health Research Centre, Dodowa, Ghana; 6 University for Health and Allied Sciences, Ho, Ghana; Sanaria,. Inc, UNITED STATES

## Abstract

**Background:**

Uncommon and rare adverse events (AEs), with delayed onset may not be detected before new drugs are licensed and deployed. The present study examined the post licensure safety of dihydroartemisinin-piperaquine (DHP) as an additional treatment for malaria in Ghana. The relationship between the incidence of AEs, treatment completion rate, participant characteristics and concomitant medications are reported.

**Methods:**

A study conducted from September 2013 to June 2014 in Navrongo, Kintampo and Dodowa health research centres in Ghana is presented. Participants had confirmed malaria and no known allergy to study drug. Patients provided informed consent and had their symptoms and results of their clinical examinations documented. Treatment with Eurartesim^®^ (20/160mg dihydroartemisinin and 40/320mg piperaquine by Sigma-Tau Incorporated) was given, according to the body weight of patients. First treatment doses were under observation but the second and third doses were taken at home except in a sub-study involving a nested cohort. Patients were contacted at Day 5 (± 2 days) either on telephone or by a home visit to document any AEs experienced. Patients were asked to report to the study team any other AEs that occurred within 28 days post-treatment. All patients in the nested cohort had electrocardiogram (ECG).

**Findings:**

A total of 4563 patients, 52.1% females and 48.2% <6 years completed the study. A total of 444 patients were enrolled into the nested cohort. About 33% had temperature ≥ 37.5°C at enrolment. Approximately 3.4% reported taking prior antimalarials, 19.4% other medications and 86% took at least one concomitant medication. Incidence of AEs was 7.6% including infections (4.6%), gastrointestinal disorders (1.0%) and local reactions at the site of venesection (0.5%). Others were respiratory disorders (0.4%) and nervous system disorders (0.3%). There were nine adverse events of special interest (AESI); itching/pruritus (7), dizziness (1), and skin lesions (1). Patients who took medications prior to enrolment had higher incidence of AEs compared with those without (9.3% vs. 6.1%; P<0.001). Statistically significant associations were found between the reported AEs and age of patients (P<0.001), their body mass index (BMI) (P< 0.001) and parasite densities (P< 0.001).

**Conclusion:**

Dihydroartemisinin-Piperaquine was well tolerated with no serious safety concerns identified. Obesity and prior enrolment medication were among significant factors associated with increased AEs reporting.

## Introduction

Systematic clinical studies in human volunteers are often carried out in order to discover the effects of new medicinal products [1, 2]. Sometimes, these new medicinal products are developed and introduced in populations on the premise that the individual and community benefits justify it. However, uncommon and rare adverse events (AEs) with delayed onset are usually not detected before such new products are deployed into the general population for widespread use [3]. Significant subpopulations with underlying medical conditions are often excluded in clinical trials but get exposed to these new products once they are introduced into the public health system [3, 4]. When new medicinal products are introduced for use in the general population, recipients are no longer critically monitored for AEs as in clinical trials. Factors such as sample size, participants’ selection, follow-ups and surrogate endpoints often limit the generalizability of findings from clinical trials. Moreover, due to health system challenges, when large numbers of the population are exposed to new medicinal products, there can be an exaggerated emergence of AEs that may undermine the usefulness of such new products [5–7]. Besides, conditions and reasons for safety monitoring may change following routine use of new medicinal products in the public health system [8, 9]. Thus, post-licensure surveillances are carried out to expand the evidence base of the characteristics of new products for which licensure has been granted [8–10].

Artemisinin-based combination therapies (ACTs) are presently recommended for the treatment of uncomplicated *falciparum* malaria. Currently, dihydroartemisinin plus piperaquine (DHP) has been made an option for the first-line treatment of uncomplicated malaria [11]. The choice of any of these ACTs has been based on the level of resistance of the artemisinin partner drug in the area [11]. The millions of doses of ACTs that are being introduced into the health system necessitate the establishment of pharmacovigilance systems in malaria endemic countries. This is essential as the weaknesses in existing health systems are likely to reduce the effectiveness and safety of these new antimalarials being introduced for large-scale use.

To contribute to post-licensure safety information on antimalarials, the INDEPTH-Network in 2009 established an effectiveness and safety platform for post licensure evaluation of newly registered antimalarials across Africa [12]. The Phase IV platform involved eight sites in Africa with health and demographic surveillance systems and well-established pharmacovigilance centres for spontaneous adverse events reporting [13].

In Ghana, three first-line artemisinin-based combination drugs are available for treatment of uncomplicated malaria—Artesunate-amodiaquine (ASAQ) and Artemether-lumefantrine (ALU) have been in the public health system for some time but Dihydroartemisinin-piperaquine (DHP) has recently been introduced and has limited pharmacovigilance safety data.

This study used the INDEPTH-Network safety-monitoring platform and data from a prospective phase IV study that evaluated clinical safety of fixed-dose dihydroartemisinin-piperaquine [13].

This study assessed the safety of DHP as an additional first-line combination treatment for acute uncomplicated malaria across three different ecological zones with health and demographic surveillance systems in Ghana.

The dihydroartemisinin (DHA) component of DHP is very rapidly absorbed with Tmax being about 1–2 hours after single and multiple dosing. The Piperaquine (PQ) component is highly lipophilic, slowly absorbed and has a Tmax of about 4–5 hours after single and repeated dose [14]. Piperaquine can accumulate in patients’ plasma after multiple doses with an accumulation factor of around three due to its slow elimination [14]. The increase in PQ concentration is more pronounced when administered with a high fat or high calorie meal and the effect is of clinical relevance due to prolonged QTc interval [14–16].

In view of the concerns about cardiotoxicity of DHP [14–16], this study investigated the safety of DHP when used under normal conditions in patients with acute signs and symptoms of uncomplicated malaria. It particularly assessed the relationship between occurrence of adverse events, study completion rate, participant characteristics and concomitant medication, and changes in QTc intervals in a subgroup of the participants.

## Materials and methods

### Study settings

**Fig 1:**
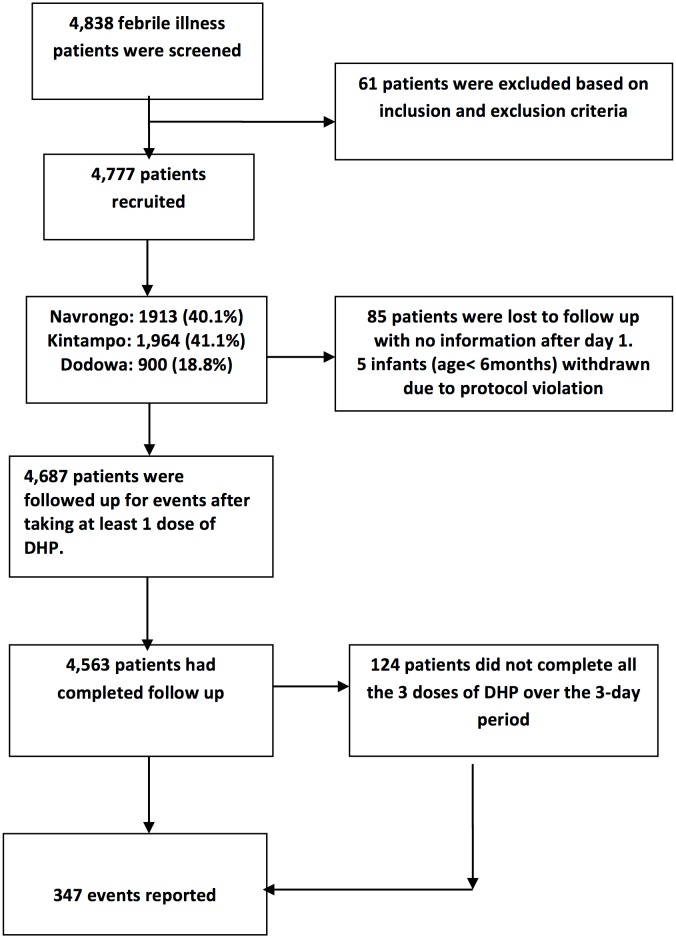
The geographical location of the three health and demographic surveillance sites in Ghana where the study was conducted. This figure is for illustrative purposes only.

The study was conducted across Ghana in three health research centres where there are on-going Health and Demographic Surveillance Systems (HDSS) Fig 1. The epidemiology of malaria and the demographic features of the three sites have been described elsewhere [17–19]. Briefly, the Dodowa HDSS is located in South-eastern Ghana covering two peri-urban districts of Shai-Osudoku and Ningo-Prampram in the Greater Accra region. It has a combined population of about 112 000, surface area of 1528.9 km^2^ and characterized by coastal savannah vegetation [17]. The Kintampo HDSS is located in the forest- savannah transitional zone in the middle belt of Ghana. The area is rural comprising the Kintampo North and South districts with about 7,162 km^2^ surface area and a combined resident population of about 143 000 [18]. The Navrongo HDSS covers two districts—the Kassena-Nankana Municipal and the Kassena Nankana West districts of northeastern Ghana. The districts are predominantly rural with an area of 1,685 km^2^, population of about 156 000 and Guinea Savannah vegetation [19].

Malaria is endemic in all the three study sites with *Anopheles gambiae s*.*l* and *Plasmodium falciparum* being the most predominant malaria vector and parasite respectively. Recent parasite prevalence estimates ranged from 5% along the coast to about 40% in northern Ghana [20]. The Ghana national malaria control programme in 2013 estimated 11.3 million malaria cases, approximately 43% of all out patient department attendances and 417 cases per 1,000 populations. The first line treatments for uncomplicated malaria in Ghana are artesunate-amodiaquine, artemether-lumefantrine and recently dihydroartemisinin-piperaquine. All the sites were part of the INDEPTH-Network Phase IV platform set up to evaluate the safety and effectiveness of antimalarial drug treatments [12].

### Study design and population

This was a prospective, observational, open-label, non-comparative, multi-site study conducted from September 2013 to June 2014 across the three sites [13]. A core set of patients were recruited and followed up as in a real world health system. Additional participants formed into a relatively smaller nested cohort constituted by patients who agreed to participate in the closer monitoring conditions were consecutively enrolled to evaluate the effect of the administration of Eurartesim^®^ on blood biochemistry, full blood count, white cell count differential count and QTc intervals. In this subset, the presence of *Plasmodia* of any species was confirmed microscopically before treatment and was intensively followed-up. Blood was taken for haemoglobin, full blood count and biochemistry at Day 1 before drug administration, Day 3 between 3–4 hours after the last dose of treatment, and Day 7. If the results were abnormal and clinically relevant, the blood examination was repeated weekly until normalization. They also had ECGs undertaken on Day 1 before drug administration, twice on Day 3 (i.e. before and 3–4 hours after the last drug administration) as well as on Day 7.

All patients visiting selected health facilities in the study sites during the study period were screened for inclusion. The description and analysis presented in this report covers mainly detailed data from the three study sites in Ghana: Navrongo, Kintampo and Dodowa health and demographic surveillance sites.

Study participation included persons of both sexes with presence or history of fever within the previous 48 hours, chills, headache, general malaise and/or loss of appetite. All eligible patients were invited to participate in the study after the necessary individual and/or parental informed consent and/or assent had been obtained. Each patient had detailed symptoms enquiry, clinical examinations and all significant conditions documented. Each patient was allowed to enter the study once and had to satisfy the selection criteria.

The inclusion criteria were as follows; uncomplicated malaria diagnosed as per WHO recommendations, age ≥6 months and weight ≥5 kg. Others were the patient’s ability to take oral medication, willingness to participate based on a witnessed signed informed consent, and access to a health facility. The patient had to agree to comply with all scheduled follow-up visits. The exclusion criteria included a known allergy to artemisinin or to piperaquine, known pregnancy, lactating women and patients with complicated malaria. All patients who were on drugs that are known to prolong the QTc interval or have antimicrobial effects or are non-sedating antihistamines or have taken a DHP in the previous four weeks were also excluded. Others included a family history of sudden unexplained death, or personal or family history of predisposing cardiac conditions for arrhythmia/QT prolongation.

### Treatment and safety assessments

Drug treatment was based on weight bands using the two available dosage strengths of Eurartesim^®^ (20/160mg and 40/320 mg DHP produced by Sigma-Tau Pharmaceuticals, Incorporated). The first dose was administered under observation to all patients in the clinic. Patients were instructed to remain in the clinic for observation for at least one hour. The second and third daily doses were self-administered at home on consecutive days and at the same time as the first dose. Those in the small nested cohort took all three treatment doses under direct observation. Patients who vomited within 30 minutes of administration of the first dose were re-treated with the same dosage, and if vomiting occurred within 30 to 60 minutes, half a dose was re-administered. Re-dosing was not attempted more than once.

Patients were contacted on Day 5 (± 2 days) either by telephone or by a home visit by trained field supervisors for assessment of their recovery status; all adverse events experienced after treatment were documented during this visit or phone call. For all instances where adverse events information collected during the telephone contact was considered incomplete, a study staff was asked to visit the patient to complete the interview. In addition, patients were asked to report to the study team any other adverse events that occurred within 28 days after drug administration. Patients whose symptoms worsened between intake of the first dose and within 28 days of study enrolment were asked to visit the nearest health facility or contact study staff using the telephone numbers on their consent form. In cases of serious and / or severe adverse events classified as of special interest, a field supervisor was detailed to bring the participant for further assessment by the study clinicians. If any patient reported a cardiac event, an ECG was performed and the trace was inspected for other abnormalities. Any patient with events deemed to be serious were cared for in line with the national standard practices.

The AESI were predefined in the study and in all situations patients were directed to the health facility for evaluation and recording of all relevant information. AESI suggestive of possible cardio-toxicity and prolonged QT were palpitations, seizures, pounding or pain in the chest area and fainting or syncope. AESI suggestive of neurotoxicity were seizures, dizziness, pins and needles sensations, visual disturbance, difficulties in coordination and tinnitus. Those suggestive of possible cutaneous reactions and phototoxicity were urticaria, angioedema, skin lesions, itching pruritus, discoloration and dermatitis.

### Electrocardiogram assessments

Patients in the nested cohort had a baseline 12-leads digitalized ECG done in triplicate. Print outs of the ECGs with at least five complexes for each lead were read by the study physicians before the first dose was administered. For each test, automatic calculation for QTcF was verified and participants with average recordings of ≥ 450ms excluded and prescribed other antimalarials. Electronic copies of all the ECGs were sent out to a central cardiac laboratory (Cardiabase) in France for further evaluation. Quality control checks included the assessment of the intra-reader and inter-reader variability. Pre-dose ECG was performed before day 3 dose was administered and patients were observed for 3 to 4 hours before the day 3 post-treatment ECGs in triplicate were performed. Participants whose day 3 pre-treatment QTc interval were ≥ 500 ms were observed for 6 hours till the QTc interval was less than 480ms before the third dose was administered. Alternate antimalarial medicines were administered if the QTc interval persistently remained ≥ 480ms. On day 7, ECGs were repeated for each participant.

### Laboratory procedures

The initial diagnosis of malaria was confirmed with malaria rapid diagnostic test as per the World Health Organization (WHO) recommendation [11]. In addition, thick blood smears were prepared on the first day, air dried, stained with Giemsa and stored for independent confirmation of presence or absence of malaria parasites. Qualified personnel not associated with the study later examined the slides in the laboratory. Patients in the nested cohort subset had full haematological, biochemical and hepatic profiles done on the first day before drug administration, on day 3 following 3–4 hours after the last dose of treatment and on day 7. If the results were abnormal and clinically significant, the blood tests were repeated weekly until normalization. Each blood sampling required about 2mL of venous whole blood.

### Data management and analysis

Data were double-entered into an on-line system using OpenClinica software, which is good clinical practice compliant. Statistical analysis were performed using STATA^®^ (version 11.2) software package. Descriptive analyses were conducted for all data. In addition, results from bivariate analysis, logistic regression, Chi-square, odds ratios are presented. Body Mass Index (BMI) calculated as weight in kilograms divided by the square of height in meters was estimated. The BMI is an indicator of the level of body fatness and is therefore used for the assessment of overweight and obesity. For adults a BMI value of 30 or more is regarded as obesity. We estimated BMI for those aged ≥18 years. Adverse events were coded and described using the Medical Dictionary for Regulatory Activities (MedDRA^®^, version 13.1) terminology. All events reported were grouped by MedDRA^®^ system organ class classification. The estimates of the incidence of AEs were based on crude rates with no causality assessment of individual cases. A two-sided 95% confidence interval (CI) was constructed for the estimates. The QTc intervals (ms) were evaluated after correcting for the heart rate with Fredericia’s formula (QTcF = QT/RR^1/3^). Descriptive analysis of the mean QTcF was done. The proportion and mean difference between the baseline and day 3 pre-dose, day 3 post-dose and day 7 were computed. The pre- and post-dose mean and median values of all the laboratory parameters were described.

### Ethics and approvals

The justification for the use of human participants for this study was to evaluate the safety of DHP when used in patients with signs and symptoms of uncomplicated malaria in a real life situation. The protocols received approvals from the Ghana health service ethics review committee and the Navrongo, Kintampo and Dodowa health research centre institutional review boards respectively. Approval was also obtained from Ghana’s foods and drugs authority. The approvals were followed by community engagement to seek the support of the communities and their leaders. The study was conducted in compliance with good clinical and laboratory practices. Written informed consent was obtained from all study participants before any study-related activity was undertaken. Parental consent was obtained for children and assent for older children (12–17 years). The study was registered with Clinicaltrials.gov (NCT02199951).

## Study findings

### Baseline characteristics

**Fig 2:**
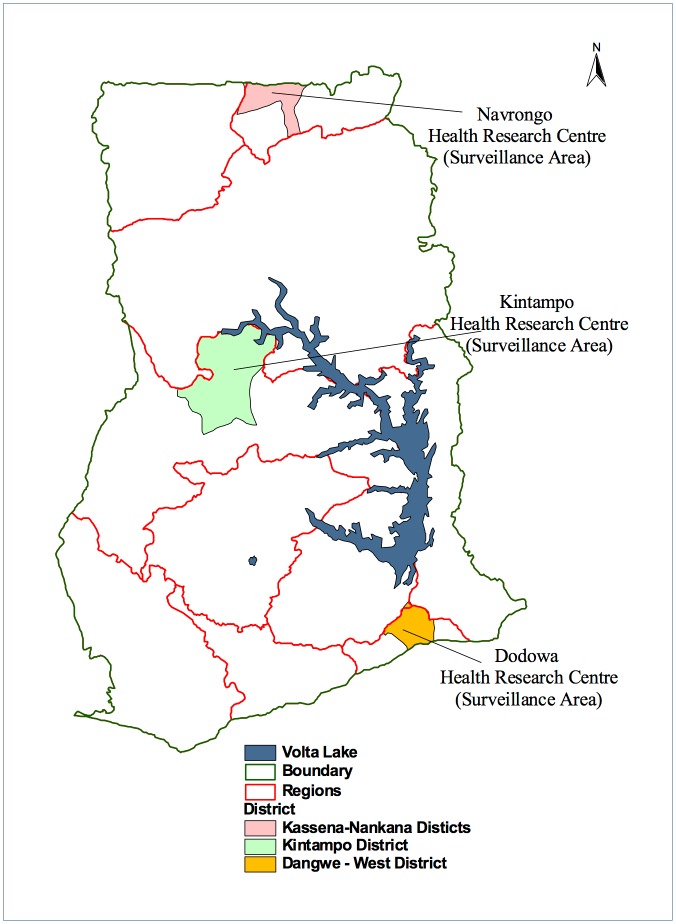
CONSORT flowchart for overall study screening, recruitment and enrolment.

A total of 4838 patients with acute febrile illness were screened, 4777 enrolled and 4563 completed the study. A total of 444 patients were enrolled into the nested cohort (Table 1). Kintampo, Navrongo and Dodowa Health Research Centres contributed 41.1%, 40.1% and 18.8% respectively. Fig 2 presents CONSORT flowchart of the study recruitments. In all 214 patients were withdrawn from the study: 39.7% due to lost to follow-up, 2.3% for selection criteria violation and 57.9% for incomplete study procedures. Among the 4563 evaluable participants, 52.1% were females, 48.2% were <6 years of age and 16.0% above >18 years of age. Site-specific completion rates were Navrongo (99.4%), Dodowa (98.2%) and Kintampo (90.3%). The average (SD) participant age, weight and height were 10.9 years (13.6), 26.1 kg (18.7) and 117.4cm (31.3) respectively. Participants from Dodowa (33.6kg; SD = 21.7) were on average heavier compared to those from Navrongo (27.8kg; SD = 19.2) and Kintampo (21.3kg; SD = 15.2). Adult patients were 23% in Dodowa, 19% in Navrongo and 8% in Kintampo. Additional background details are in Table 1.

**Table 1:** Background clinical and demographic characteristics of the study participants.

Characteristics	Attributes	Navrongo	Kintampo	Dodowa
Recruitment	Total (%)	1901 (41.7)	1778 (39.0)	884 (19.3)
	Nested (%)	274 (61.7)	15 (3.4)	155 (34.9)
Age of Participants (years)	Average (SD)	12.6(15.4)	7.6(9.3)	14.7(15.6)
Weight of Participants (kg)	Average (SD)	27.8(19.2)	21.3(15.2)	33.6(21.7)
Sex of participants, n (%)	Males	908 (47.8)	867 (48.8)	410 (46.4)
	Female	993 (52.2)	911 (51.2)	474 (53.6)
Age in years, n (%)	< 6	837 (44.0)	1057 (59.5)	307 (34.7)
	6–12	522 (27.5)	488 (27.5)	254 (28.7)
	13- <18	172 (9.1)	84 (4.7)	113 (12.8)
	≥ 18	370 (19.5)	149 (8.4)	210 (23.8)
Fever (T ≥ 37.5°C), n (%)	Yes	555 (29.2)	721 (40.6)	222 (25.1)
	No	1346 (70.8)	1057 (59.5)	662 (74.9)
^1^Parasite density/μL, n (%)	<50	53 (4.0)	13 (1.0)	24 (3.3)
	50–499	288 (21.8)	105 (7.7)	77 (10.5)
	500–4,999	223 (16.9)	206 (15.2)	205 (27.8)
	5000–49,999	556 (42.1)	377 (27.6)	183 (24.8)
	≥ 50,000	200 (15.2)	659 (48.5)	248 (33.6)
Ingestion of antimalarials within the last 4 weeks, n (%)	Males	37 (4.1)	23 (2.7)	8 (2.0)
	Females	45 (4.5)	39 (4.3)	4 (0.8)
Ingestion of any medication within the last 2 weeks, n (%)	Males	144 (15.6)	159 (18.3)	131(32.0)
	Females	159 (16.0)	159 (17.5)	134 (28.2)

^1^The discrepancy between the total number enrolled and the number with parasite density is that the initial diagnosis of malaria was confirmed with malaria rapid diagnostic test

About 33% (1498/4563) of patients had axillary temperature ≥ 37.5°C at enrolment and this varied across the sites; Kintampo (40%), Navrongo (29.2%) and Dodowa (25.1%). Age specific fever prevalence were; < 6 years (43.2%), 6–12 years (31.0%), 13–18 (21.7%) and >18years (13.1%). The frequency of baseline symptoms were fever (26%), anorexia (11%), headache (11%), vomiting (10%), cough (9%), weakness (9%), abdominal pain (7%), muscular pain (5%), diarrhea (4%) and nausea (4%). About 12% of all patients had parasite density less than 500/μL and 24% > 50,000 /μL. Geometric mean parasite density (95%CI) by site was 4752/μL (4185, 5396) in Navrongo, 25503/μL (22558, 28833) in Kintampo and 7995 /μL (6706, 9533) in Dodowa. About half of the patients from Kintampo (48.5%) had parasite density of >50,000 /L compared to 15% in Navrongo and 33.3% in Dodowa. Age specific parasite density (95% CI) was 23802/μL (21426, 26441) in < 6year olds, 9897/μL (8544, 11464) in 6–12 year olds, 2434 /μL (1804, 3285) in 13–18 year olds and 1540/μL (1264, 1876) those >18 years (Table 1).

Approximately 3.4% (156/4563) of patients reported taking antimalarials within the last four weeks prior to enrolment. The prevalence was highest in Navrongo 4.3% (82/1901), followed by Kintampo 3.5% (62/1778) and Dodowa 1.4% (12/884). About 70% (106/156) of all antimalarials taken prior to enrolment were in children < 6years of age. The age specific prevalence was <6years (4.8%), 6-12years (1.9%), 13–18 years (1.1%) and >18years (3.0%). The prevalence in females and in males were similar (3.7% vs. 3.1%; P = 0.26). Approximately 19.4% (95% CI 18.2, 20.5) of patients reported taking other medications prior to enrolment. The site-specific prevalence was Navrongo (15.9%), Kintampo (17.8%) and Dodowa (30%). About 20% were in children < 6 years, 19.0% in females and 20% in males.

### Concomitant medication

**Fig 3:**
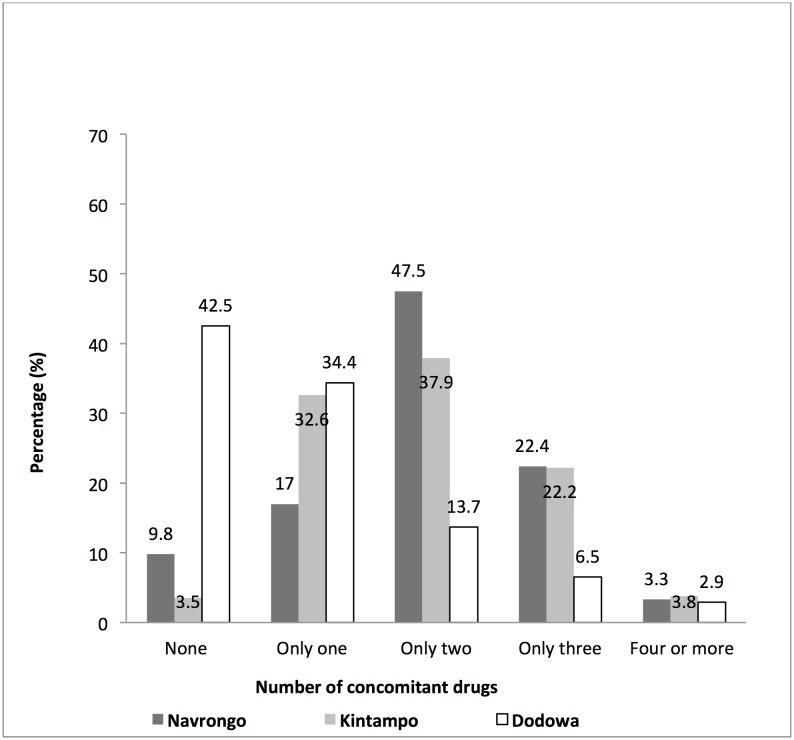
The total number of concomitant medication per study site taken by study participants during the study period.

**Fig 4:**
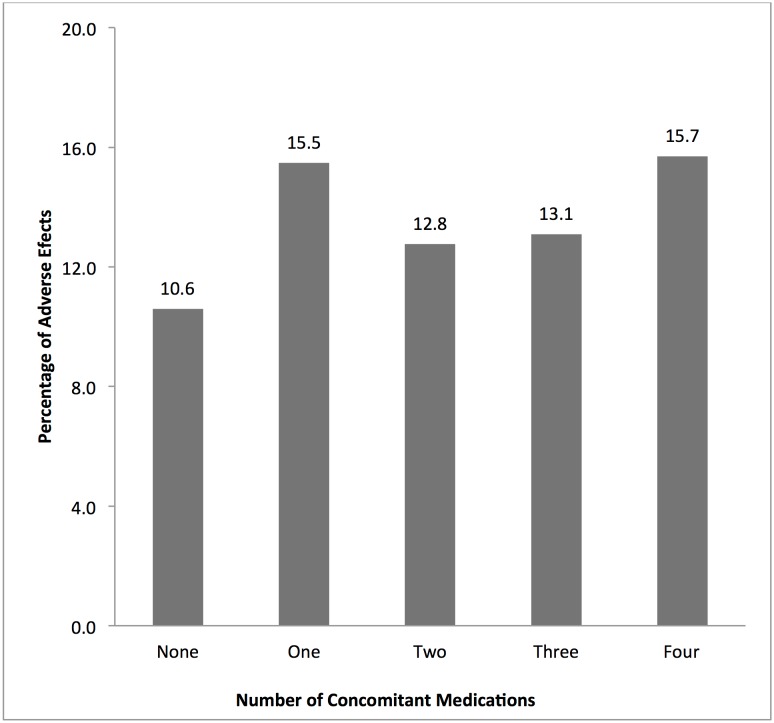
The relationship between the incidence of reported adverse events per number of concomitant medications reportedly taken by the study participants during the period.

Approximately 86% (3938/4565) of the participants took at least one concomitant medication during the study. The proportions of patients who took concomitant medications per site were Navrongo (90.2%), Kintampo (96.5%) and Dodowa (57.5%). Fig 3 shows the percentage of patients by the number of concomitant medications taken per study site. The proportions of females and males who took concomitant medication were comparable (85.8% vs. 86.9%; P> 0.05). There were significant differences among the four categorized age groupings on the use of concomitant medications (χ^2^ = 18.7; P< 0.0001). Children aged <6 years had the highest (88.2%) compared to those aged 6–12 years (83.4%), 13–18 years (87.8%) and above 18 years (84.6%). Fig 4 shows the percentage of AEs per number of concomitant medications taken by the study participants.

### Incidence of AEs

Table 2 presents the total number of AEs per each organ system, the incidence rate per 10000 population and by study site. The total incidence of AEs was approximately 7.6% (347/4563). The five commonest AEs recorded were infections and infestations (4.6%), gastrointestinal disorders (1.0%), and general disorders and local reactions at the site of venesection (0.5%). Others were respiratory, thoracic and mediastinal disorders (0.4%) and nervous system disorders (0.3%). All systems recorded at least one adverse event. Incidence of AEs varied across the three sites (χ^2^ = 18.7; P <0.0001), ranging from 9.2% (95% CI 7.9, 10.5) in Navrongo, 8.4% (95% CI 6.6, 10.3) in Dodowa to 5.5% (95% CI 4.4, 6.6) in Kintampo. Infection and infestations, and gastrointestinal disorders were the leading incidence of AEs reported in all sites Table 2. Incidence rate per 10000 population ranged from 551 in Kintampo to 921 in Navrongo. The number of organ systems that reported no AEs was three in Navrongo, six in Kintampo and ten in Dodowa. There were nine AESI: seven were due to itching and pruritus, and one each from dizziness and skin lesion. The incidence of AESI was therefore estimated to be 20 cases per 10 000 population. Three AESI were in the age group under six years; two in the 6–12 age group and four were in those above 18 years of age. There were three in males and six in females and three from Navrongo, six from Dodowa and none from Kintampo.

**Table 2:** Number and reported adverse events per 10 000 population by study site.

Adverse Events	Number (Incidence /10,000)			
	Total N = 4563	Navrongo N = 1901	Kintampo N = 1778	Dodowa N=884
Blood and lymphatic system disorders	5 (11)	2 (11)	2 (11)	1 (11)
Cardiac disorders	2 (4)	0 (0)	2 (11)	0 (0.0)
Congenital, familial and genetic disorders	2 (4)	0 (0)	2 (11)	0 (0.0)
Eye disorders	3 (7)	2 (11)	1 (6)	0 (0.0)
Gastrointestinal disorders	47 (103)	10 (53)	13 (73)	24 (271)
General disorders and local reactions at the site of venesection	21 (46)	10 (53)	3 (17)	8 (90)
Immune system disorders	1 (2)	1 (5)	0 (0)	0 (0)
Infections and infestations	212 (465)	123 (647)	62 (349)	27 (305)
Injury, poisoning and procedural complications	2 (4)	2 (11)	0 (0)	0 (0)
Metabolism and nutrition disorders	2 (4)	2 (11)	0 (0)	0 (0)
Musculoskeletal and connective tissue disorders	9 (20)	5 (26)	0 (0)	4 (45)
Nervous system disorders	13 (28)	3 (16)	4 (22)	6 (68)
Pregnancy, puerperium and perinatal conditions	1 (2)	1 (5)	0 (0)	0 (0)
Psychiatric disorders	2 (4)	2 (11)	0 (0)	0 (0)
Renal and urinary disorders	2 (4)	1 (5)	1 (6)	0 (0)
Reproductive system and breast disorders	1 (2)	0 (0)	1 (6)	0 (0)
Respiratory, thoracic and mediastinal disorders	10 (22)	5 (26)	4 (22)	1 (11)
Skin and subcutaneous tissue disorders	12 (26)	6 (32)	3 (17)	3 (34)
Total	347 (76)	175(921)	98 (551)	74 (837)

### AEs and participants’ attributes

**Fig 5:**
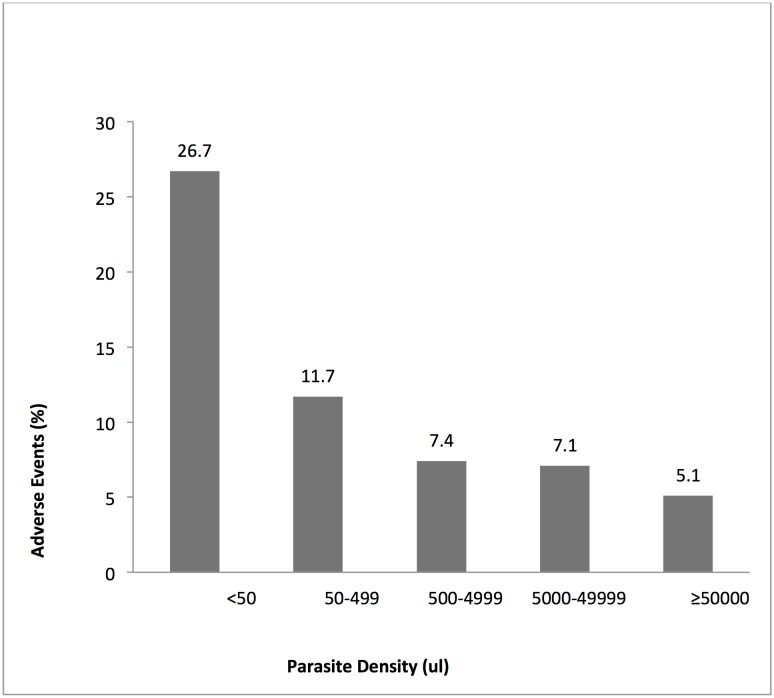
This figure shows a negative correlation between the incidence of reported adverse events and categories of malaria parasite density among the study participants.

**Fig 6:**
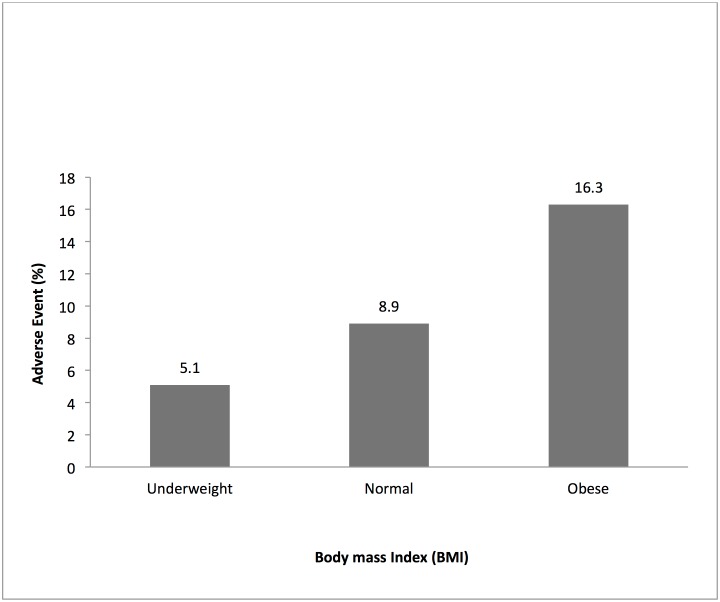
The relationship between the incidence of adverse events and body mass index as characterized as underweight, normal and obese among the study participants who were eighteen years of age and above.

Bivariate analysis showed that there was no significant difference in the incidence of AEs by sex (χ^2^ = 2.9; P> 0.05) and by concomitant medication (χ^2^ = 3.5; P > 0.05). Patients who took other medications prior to enrolment had higher incidence of AEs compared with those who did not (9.3% vs. 6.1%; P<0.001). Statistically significant heterogeneity of AEs was found between the study treatment and different age groups (χ^2^ = 22, p<0.0001), body mass index (χ^2^ = 18.9; P< 0.0001) (Fig 5) and malaria parasite density (χ^2^ = 67.8; P< 0.0001) (Fig 6). There was a positive correlation between body mass index and AEs (χ^2^ for trend = 17.1; P< 0.0001) (Fig 5) but parasite density significantly correlated negatively with the AEs (χ^2^ for trend = 20.3; P< 0.0001) (Fig 6). Logistic regression analyses showed that Kintampo site compared with Dodowa (OR = 0.37, 95% CI 0.16, 0.84), obese compared to underweight patients (OR = 3.17, 95%CI 1.49, 674) and prior enrolment medication (OR = 2.23, 95%CI 1.28, 3.89.) were the significant factors associated with AEs. Relationship between AEs and participants’ attributes is presented in Table 3.

**Table 3:** Reported incidence of adverse events by patient attributes.

Characteristics	Attributes	n (N)	% (95%CI)	χ^2^ (Pvalue)
Enrolment	Main	264 (4119)	6.4 (5.6, 7.2)	86.1(0.0001)
	Nested	83 (444)	18.7 (15.1, 22.6)	
Study Site	Navrongo	175(1901)	9.2 (7.9, 10.5)	18.7(< 0.001)
	Kintampo	98 (1778)	5.5 (4.4,6.6)	
	Dodowa	74 (884)	8.4 (6.6, 10.3)	
Sex of Patients	Males	151 (2185)	6.9 (5.8, 8.0)	2.9(0.094)
	Females	196 (2378)	8.2 (7.1, 9.3)	
Age groups (years)	< 6	157 (2201)	7.1 (6.0, 8.2)	22.6(<0.001)
	6–12	82 (1264)	6.5 (5.1, 7.9)	
	13–18	22 (369)	6.0 (3.7, 8.8)	
	>18	86 (729)	11.8 (9.5,14.3)	
BMI (age 18 ≥ years)	Under	19 (370)	5.1 (3.1, 7.9)	18.9(<0.001)
	Normal	57 (641)	8.9 (6.8, 11.3)	
	Obese	30 (184)	16.3 (11.2, 22.4)	
Concomitant medications during treatment	Yes	288 (3938)	7.3 (6.4, 8.1)	3.5(0.062)
	No	59 (625)	9.4 (7.2, 12.0)	
Prior enrolment other medications	Yes	82 (886)	9.3 (7.4, 11.3)	11.2(0.001)
	No	225 (3677)	6.1 (5.3, 6.9)	
Parasite density (μL)	< 50	24 (90)	26.7 (17.8, 37.0)	67.8(<0.001)
	50–499	55 (470)	11.7 (8.9, 14.9)	
	500–4999	47 (634)	7.4 (5.4, 9.7)	
	5000–49999	80 (1116)	7.1 (5.6, 8.7)	
	≥ 50000	56 (1107)	5.1 (3.8, 6.5)	

### ECG findings

Change in QTcF values in each individual patient were assessed by comparing the number of patients who had increases and decreases in the parameters. In all, findings from 378 patients on day 3 were found to have increased, 90 were decreased and 9 had no change from the baseline findings. The mean QTcF at baseline (day 1) was 394.8ms (95%CI 392.7, 396.8). There was no significant difference in the QTcF between males (393.8ms) and females (395.6 ms) at baseline (-1.8 (95% CI -5.9, 2.3). The mean QTcF (95% CI) on day 3 pre-dose, day 3 post-dose and day 7 were 409.3ms (407.4, 411.1), 422.9ms (420.9, 425.0) and 399.2ms (397.3, 401.0) respectively. Compared to the baseline value there were significant changes in mean QTcF in day- 3 pre-dose -14.5ms (-16.3, -12.7), day-3 post-dose -28.2ms (-30.3,-26.0) and day-7 post-enrolment -4.5ms (-6.1,-2.8). For three patients who had QTcF above the 500ms cutoff value, one patient had QTcF of 509ms on day-3 post-dose and the same patient had QTcF of 516ms on day-7 post-enrolment. These were however not clinically significant and resolved after follow-up. The number of patients with QTcF prolongation > 60ms from their baseline were seven on day-3 pre-dose, 38 on day-3 post-dose and only one on day-7 post-enrolment and none of them was clinically significant.

### Laboratory results

Patients who were enrolled into the nested arm of the study had safety evaluation done for their laboratory parameters. Of the 444 patients who provided blood samples 61.7% (274), 34.9% (155) and 3.4% (15) were recruited from the Navrongo, Dodowa and Kintampo sites respectively. Approximately 46% (95% CI 41.2 50.7) were males. Change in parameter values in individual patient, before and after treatment was assessed by comparing increases and decreases in the parameter. The findings were as follows; haemoglobin (16.5% vs. 80.2%; p<0.0001), total RBCs (20.2% vs. 79.1%; p<0.0001), total WBCs (41.7% vs. (56.1%; p<0.0001), total bilirubin (32.8% vs, 67.2%; p<0.0001) and total Chloride (63.4% vs. 34.2%: p<0.0001). Others were total alanine transaminase (41.7% vs. 57.2%; p = 0.101), total aspartate transaminase (42.9% vs. 55.6%; p = 0.146) total potassium (48.4% vs. 47.0%; p = 0.192) and total creatinine (46.8% vs, 51.5.0%; p = 0369). In addition, the means across the entire subgroup- before and after treatment were assessed as follows; total mean haemoglobin g/dL (10.7 vs. 9.9; p< 0.0001); total red cells x 10^6^/μL (4.2 vs. 3.9; p< 0.0001) and total white cells x 10^6^/μL (7.6 vs. 6.8; p< 0.0001). For the clinical chemistry, the respective mean baseline (day 0) values compared to day 3 estimates were total bilirubin, μmol/L (18.9 vs 7.0; p< 0.0001), alanine transaminases, U/L (26.9 vs. 25.9; p = 0.61), aspartate transaminases, U/L (33.1 vs. 31.3; p = 0.28 and creatinine, μmol/L (53.5 vs. 53.3; p = 0.89). Others were blood urea nitrogen, μmol/L (4.6 vs. 2.9; p< 0.0001), chloride, μmol/L (105.3 vs. 108.2; p = 0.0064 0.05) and potassium, μmol/L (3.9 vs. 3.9; p = 1.00). The changes in the biochemical parameters were not clinically significant.

## Discussion

The study determined additional safety data on DHP as a first line treatment for uncomplicated *falciparum* malaria in Ghana and the capacity of the three sites to undertake post-licensure surveillance of new medicinal products [12].

Approximately 95% of all the enrolled patients completed the study as planned. High treatment completion rate in a clinical trial suggests treatment acceptability given that participants are more likely to drop out of a study where adverse events are many and intolerable. The treatment completion rate in this study is similar to previous reports that showed the effectiveness of DHP for malaria in endemic countries [16, 21, 22]. Further analysis carried out on type and frequency of AEs reported did not find anything significant. The main reasons given for dropping out in this study was loss to follow-up, selection criteria violation and incomplete study procedures and were not different from what was earlier reported [16, 21, 22]. The frequencies of AEs documented were mild in severity, non-serious in nature and consistent with those often expected in patients with acute malaria [16, 22]. Though the incidence of AEs varied across organ systems, the commonest remained the same. There were nine AESI, which presented no serious or severe outcomes consistent with recent reviews on DHP safety and acceptability [21,22].

Patients taking antimalarials prior to being enrolled were mostly children with similar characteristics across sites and this did not influence the incidence of AEs. Four out of every five participants took at least one additional medication during the study participation.

Taking additional medication during study participation was not associated with higher incidence of AEs compared to those who took medications prior to enrolment. Patients who had taken medications prior to enrolment were two times more likely to report AEs. Though self-medication increases access to medication and relief for acutely sick persons, it can also lead to serious drug interactions and AEs [23]. Self-medication needs to be considered as exclusion criteria to minimize the incidence of AEs in clinical trials.

Higher incidence of AEs was observed in the rural settings compared to the semi-urbanized settings. This was expected, as rural residents are more likely to have lower incomes, less access to health care and more likely to self-medicate [23, 24]. They are also more prone to infections and infestations, which promotes self-medication. This can lead to increased drug interactions and AEs resulting from incorrect choice, dosage and administration of medicines [23]. Further studies on the rationale for prior self-medication among rural residents could help reduce adverse events and improve treatment acceptability and adherence. Taking concomitant medication was not sex dependent in this study. Statistically significant heterogeneity was found between AEs and different age groups and as expected children were reported to have the highest proportion of concomitant medication probably because they were likely to have multiple infestations and receive medication from their caregivers. As children are less likely to communicate any adverse events, the implication will be that, for children any excessive intake of prior and concomitant medication that is not excluded in the study selection process may pose additional risk to their health and the study outcomes.

Therapeutic drugs often demonstrate negative side effects on concomitant treatment hence the need to collect data on such drugs over time. Concomitant medications can either increase or decrease the metabolism of an investigational drug. When treatment metabolism is reduced as a result of concomitant medication, the resultant effects will be symptoms of over dosage even if the patient has taken the treatment as prescribed. Conversely, if there is increased metabolism, it may lead to faster excretion of the active metabolite of the investigational drug and it may not be able to reach its peak and potency at the prescribed dosage [25, 26].

Statistically significant heterogeneity was found between AEs and body mass index with a positive correlation between them. This suggests a potential for higher plasma concentrations of DHP in obese persons and therefore its administration to patients based on weight should be done with circumspection. Piperaquine as a lipophilic drug with slow elimination and long half-life [25, 26] can easily accumulate in obese patients and induce more AEs as was observed.

Safety evaluation of DHP by electrocardiogram showed that, compared to the baseline value, there were significant changes in mean QTcF post-enrolment. For patients who had QTcF above the 500ms cutoff value however, only one person had higher QTcF on both day-3 postdose and day-7 post-enrolment. Only one patient with QTcF prolongation > 60ms had such on day-7 post-enrolment and none of them was clinically significant. These few and clinically insignificant QTcF changes are consistent with earlier reports [14,15], which suggests that the combination may be associated with rare cardiotoxicity [14, 15, 25, 26].

### Limitations

It should be noted that while this was a large study, the sample size may still not have been enough to detect rare serious adverse events. Secondly, some of the findings as for example some of the AEs may not have been properly assessed particularly in very young children. Further, the site- specific variation in AE incidence could reflect ascertainment bias and the fact that some patients who had higher parasite densities could be more symptomatic of their malaria.

## Conclusion

This study has added additional safety data to show that DHP is safe even in challenged and post licensure settings. No significant serious AEs or ASEI were documented. The findings indicate that in rural settings, obese patients and pre-enrolment medication were the significant factors associated with AEs. This study demonstrated the capacity of the three research sites to undertake phase four studies to detect rare AEs of new drugs over a much larger patient population. Further technical support and collaboration will enable the sites to conduct similar studies for other drugs and vaccines across Ghana and beyond.

## Supporting information

S1 Trend Checklist (PDF)

S1 Protocol (PDF)
