# Supplementary material for: Post-licensure safety evaluation of dihydroartemisinin piperaquine in the three major ecological zones across Ghana
Source: PLoS One. 2017 Mar 30;12(3):e0174503. doi: 10.1371/journal.pone.0174503 (PMC5373525; doi:10.1371/journal.pone.0174503)
Supplement: S1 Protocol — (PDF) [file pone.0174503.s002.pdf]

OBSERVATIONAL PHASE IV STUDY PROTOCOL

PRODUCT: Eurartesim®
(dihydroartemisinin/piperaquine)

OBSERVATIONAL STUDY TO EVALUATE THE CLINICAL SAFETY AFTER INTRODUCTION OF THE FIXED DOSE ARTEMISININ-BASED COMBINATION THERAPY EURARTESIM® (DIHYDROARTEMISININ/PIPERAQUINE [DHA/PQP]) IN PUBLIC HEALTH DISTRICTS IN BURKINA FASO, MOZAMBIQUE, GHANA AND TANZANIA

STUDY NUMBER: INESS 02/2012

STUDY NAME: Introduction of Eurartesim® in Burkina Faso, Mozambique, Ghana and Tanzania

VERSION 3.1 DATE: 02/12/2013

NAMES AND ADDRESSES:

**PRINCIPAL INVESTIGATOR**	Name :<br>Address:	<u>**Prof**</u>. Fred N. Binka<br>INDEPTH-Network<br><u>**30 & 40 Mensah Wood Street, East Legon**</u><br><u>**PoBox KD 213, Kanda**</u><br><u>**Accra**</u><br><u>**GHANA**</u><br>Fred.binka@indepth-network.org
**SPONSOR**	Name:<br>Address:	INDEPTH-Network<br><u>**30 & 40 Mensah Wood Street, East Legon**</u><br><u>**PoBox KD 213, Kanda**</u><br><u>**Accra**</u><br><u>**GHANA**</u>

TABLE OF CONTENT

1. SUMMARY 6

2. LIST OF ABBREVIATIONS..14

3. INTRODUCTION AND RATIONALE 15
3.1 Summary of current situation..15
3.2 Eurartesim® 15
3.3 Malaria in Burkina Faso, Mozambique, Ghana and Tanzania..16
3.4 Project rationale..19
3.5 Proposed Sites participating to the study 19

4. STUDY OBJECTIVES..19
4.1 Primary objective..19
4.2 Secondary objectives..19

5. DESCRIPTION OF THE STUDY 20
5.1 Description of the study design..20
5.2 Study duration..22
5.3 Drug prescription and dosing 22
5.4 Evaluation criteria 23

6. STUDY POPULATION AND PATIENT SELECTION..24
6.1 Sample size..24
6.2 Selection criteria..24
6.2.1 Inclusion criteria 24
6.2.2 Exclusion criteria 24
6.3 Enrolment procedure..25
6.3.1 Investigational Sites 25
6.3.2 Patient selection..25
6.3.3 Patient numbering 25

7. STUDY PROCEDURE AND DATA COLLECTION..26
7.1 Visit planning 26
7.2 Collected data 26
7.2.1 Registry at the HDSS centre 26
7.2.2 Examination and data collection at inclusion..27
7.2.3 Follow-up visits/contacts and clinical tolerability follow-up questionnaire 27
7.3 Patients who did not receive follow-up visits/contacts..29
7.3.1 Premature discontinuation before drug intake..29
7.3.2 Premature treatment discontinuation..29
7.3.3 Patient lost to follow-up..29
7.4 Thick blood smear logistics..30
7.5 ECG Recording..30
7.6 Vital signs and Physical examination..30
7.7 PK sampling 30

8. SUBJECT SAFETY 31
8.1 Training 31
8.2 Monitoring of adverse events 31
8.3 Definition of Adverse Event and Serious Adverse Event 32
8.4 Obligation of AE notification 32
8.5 Adverse event of special interest 33
8.6 Pregnancy 35
8.7 Sponsor obligations 35

9. STATISTICAL METHODS 35
9.1 Analysis of data at study entry 36
9.2 Analysis in the whole sample 36
9.3 Determination of sample size 37

10. DATA MANAGEMENT 37
10.1 Collection and validation of data 37
10.2 Quality control of data on site 38
10.3 Quality control of the preparation of thick blood smear slides 38
10.4 Quality control of thick blood smear sample reading 39
10.5 Data-entry 39
10.6 Data cleaning and data base locking 39

11. TASKS AND RESPONSIBILITIES 40
11.1 Local Pharmacovigilance Monitoring Committee 40
11.3. Responsibilities of the Scientific Review Panel 40
11.4. Responsibilities of the clinical staff of the health facilities 41
11.5. Responsibilities of the Sponsor 42
11.6. Ethical Standards 42
11.7. Regulations 42
11.8. Data protection 42
11.9. Insurance 42
11.10. Confidentiality agreement 43
11.11. Record keeping 43
11.12. Premature termination of the study 43
11.13. Competent authority inspections 43

12. PROTOCOL AMENDMENTS 44

13. DOCUMENTATION AND UTILISATION OF STUDY RESULTS 44
13.3. Properties and use of the study data and results 44
13.4. Publications 44

14. REFERENCES 46

15. ANNEXES 47

STUDY PROTOCOL AGREEMENT FORM

I, undersigned, …………………………………………………………………….,
Principal Investigator at ……………………………………………………….....,
Hereby certify that I have examined the protocol of the above referenced study for INESS and that I have thoroughly discussed the objectives of this study as well as the contents of this protocol with the sponsor's representatives.

I agree to keep the contents of this protocol confidential and not to disclose it to a third party and to use it only for the purposes of this study.

I agree to perform this study according to this protocol and to meet the objectives, to comply with the ethical rules and to ensure patient safety.

I have well understood that in case the Sponsor would decide to prematurely end or to suspend this study at any time and for any reason, I would be informed of this decision in writing. Conversely, in case I would decide to disrupt the conduct of this study, I commit to immediately inform the Sponsor of this decision in writing.

FOR THE SPONSOR

NAME: Fred N. Binka

ADDRESS: INDEPTH-Network
30 & 40 Mensah Wood Street, East Legon
PoBox KD 213, Kanda
Accra, GHANA

DATE: SIGNATURE: ________________________________

FOR THE PRINCIPAL INVESTIGATOR

NAME:

ADDRESS:

DATE: __________________________ SIGNATURE: ______________________________

1. SUMMARY

PRODUCT: Eurartesim® **Study N°: INESS 02/2012**

**Title**	Observational study to evaluate the clinical safety after introduction of the fixed-dose Artemisinin-based Combination Therapy Eurartesim® (dihydroartemisinin/piperaquine [DHA/PQP]) in public health districts in Burkina Faso, Mozambique, Ghana and Tanzania.
**Location of the study**	Study will be performed in several public health facilities in up to **7** selected HDSS centres (Health and Demographic Surveillance **Systems**) within the HDSS in Burkina Faso (**2**), Mozambique (1), Ghana (3), and Tanzania (**1**), where Eurartesim® will be used as first-line treatment of uncomplicated malaria episodes.
**Objectives**	**Primary**:<br>Evaluate the safety of Eurartesim® when used under usual conditions in **about** 10,000 patients with signs and symptoms of uncomplicated malaria confirmed by a parasitological diagnosis (Microscopy/Rapid Diagnostic Test) or, when this test is not available, by WHO diagnostic criteria.<br><br>**Main Secondary:**<br>Intensive assessment of a nested subset of **about** 1,000 patients to evaluate the effect of the administration of Eurartesim® on blood biochemistry, full blood count, WBC differential count and QTc intervals. QTc interval and piperaquine concentration relationship will also be investigated in selected centers.<br><br>**Other Secondary objectives**:<br>Although it is expected that the vast majority of patients will be infected with *P. falciparum,* comparisons of the clinical tolerability of *Plasmodium falciparum* infected patients versus patients infected with other *Plasmodia*, as confirmed by the thick blood smear results, will be carried-out in the nested subset of **about** 1,000 patients.<br>Assessment of the relationship between the occurrence of Adverse Events and the administration of concomitant medications will also be evaluated in the subset of **about** 1,000 patients.
**Design and duration of the study**	Observational, non-comparative, longitudinal study. All patients visiting Health facilities in the HDSS areas and for whom a diagnosis of uncomplicated malaria (according to the WHO criteria) is suspected or confirmed by a parasitological diagnosis (Microscopy/Rapid Diagnostic Test) and who have signed informed consent/assent (a parent/guardian for children below 18 years old) will be included in the study.<br><br>In the subset of 1,000 patients, the presence of *Plasmodia* of any species will be confirmed microscopically **before treatment.**<br><br>A thick blood smear will be prepared for further microscopic diagnosis in all the patients. Eurartesim® tablets will be prescribed to the patients **enrolled in the main group** (or to the parents/guardians, if the patients are children) meeting the protocol

	inclusion criteria before the results of the thick blood smear are known.<br><br>The patients **<u>in the main group</u>** will be contacted at Day 5 (± 2 days), in order to capture recovery status and all the experienced adverse events. A visit by the community health agent will be scheduled on all the cases in which the information collected during the telephone contact should be considered incomplete or unreliable ones. Special procedures will be followed in case of serious and/or severe adverse events and events classified of special interest (see specific section).<br><br>The subset of 1,000 patients will be intensively followed-up. These patients will have haematology (Hb and full blood counts (RBC, WBC and differential count)) and standard biochemistry (BUN, Creat, ALT/AST, Bilirubine, electrolytes (K^+ and Cl^-)) undertaken at Day 1 (before drug administration), Day 3 (3-4 hours after the last dose of treatment), and Day 7. If the results are abnormal and clinically relevant, the blood examination will be repeated until normalization.<br><br>**<u>Each blood sampling for both haematology and biochemistry requires about 2mL of venous whole blood. This makes up for a total amount of about 6mL of venous whole blood to be taken from each participant in the nested group for the entire study period at three time points.</u>**<br><br>In all the 1,000 patients, a plasma sample will be collected on Day 1 (before drug administration), twice on Day 3 (i.e. before and 3-4 hours after the last drug administration) as well as on Day 7 to assess plasma PQ concentration. From such blood drawings and before centrifugation, three drops of whole blood will be spotted on filter papers. These filter papers will be utilized to determine whole blood piperaquine concentration with the Dry Blood Spot methodology (if a validated analytical method for such determination will be settled up at the time of the study course). ECGs will be undertaken on Day 1 (before drug administration), twice on Day 3 (i.e. before and 3-4 hours after the last drug administration) as well as on Day 7 (ECG on Day 1 and Day 3 after last drug administration will be collected in triplicate); safety information will be collected at all these visits.<br><br>**<u>About two (2) mL of venous whole blood samples will be collected from all of the 1000 subset patients (using direct venipuncture) into 5 mL tubes containing lithium heparin or EDTA depending on site specific procedures. This will be done at every PK time point making up for a total amount of 8 mL to be taken from each participant in this subgroup over the entire study period.</u>**<br><br>**<u>NB: In the subset group of about 1,000 patients, a total amount of up to 14mL of venous whole blood will be taken from each participant over the entire study period (About 6mL for haematology and biochemistry and 8mL for PK sampling).</u>**<br><br>If the QTcF (QT corrected by Fridericia's formula) value assessed on Day 3 before last dose is above 500 ms, Eurartesim® should be withheld until QTcF returns below 480 ms within 6 hours. Thereafter, the Eurartesim® cycle may be completed under frequent QTc monitoring based on medical judgment. If the QTcF does not return below 480 ms within 6 hours, another antimalarial therapy should be considered.<br><br>The occurrence of any adverse events will be solicited from the subset of 1,000 patients on Days 3 and 7 following administration of Eurartesim® as well as in any

	additional visits.<br><br>All patients in the study with a cardiac event of special interest (see below) will have an ECG erformed.<br><br>Patients will be asked to report to the health facility or to the HDSS if any adverse event occurs after Day 5 (± 2 days) contact and within 28 days after the start of Eurartesim® intake.<br><br>Female patients will be encouraged to communicate to the study team if they get pregnant within a period of two months after the start of the Eurartesim® treatment. In these cases, information on the evolution of the pregnancy will be collected at 3, 6, 9 months and after the delivery (6 and 14 weeks). Information on the drugs taken during the pregnancy as well as AEs/SAEs/AESIs and the health status of the newborn/s will be collected.
**Drug prescription and dosing**	Patients will be instructed to take Eurartesim® with a dose regimen of one administration every 24 hours over a period of three days, i.e. at Day 1, then after 24 hours (Day 2) and after 48 hours (Day 3) from the first administration.<br><br>The dose will be based on patient body weight. Two strengths of Eurartesim® will be provided to facilitate the dosing in children and adults: 20/160mg and 40/320mg of DHA and PQP respectively.<br><br>The patients will be instructed to take Eurartesim® with water, at least three hours before or three hours after food intake (i.e. three hours after the previous food intake with no food intake for the following three hours after Eurartesim® administration).<br>To facilitate drug administration in small children, tablets will be crushed on a spoon and given with water. If vomiting occurs within 30 min from drug administration, dose will be re-administered. If vomiting occurs within 30 to 60 min, half a dose have to be re-administered. Re-dosing should not be attempted more than once.
**Population**<br>Selection criteria	**Inclusion Criteria**<br>• Uncomplicated malaria (*Plasmodia* of any species) diagnosed as per national policies and in line with WHO recommendations (a history of fever in the previous 24 h and/or the presence of anaemia, for which pallor of the palms appears to be the most reliable sign in young children). Confirmation of malaria by a parasitological diagnosis (Microscopy/RDT) is encouraged but its absence does not prevent patients from being enrolled.<br>• Age ≥6 months and weight ≥5 kg.<br>• Capability of taking an oral medication.<br>• Ability and willingness to participate based on signed informed consent (a parent or a guardian has to sign for children below 18 years old), or on verbal consent given in front of a witness signing the informed consent, and access to health facility. The patient has to comply with all scheduled follow-up visits.<br><br>**Exclusion Criteria**<br>• Known allergy to artemisinin or to piperaquine.<br>• Known pregnancy.<br>• Lactating women should be excluded if other anti-malarial treatments are available

<table>
<tr><td>Number of patients

Number of sites</td><td>

Complicated malaria.
Taking medicinal products that are known to prolong the QTc interval. These include (but are not limited to):
• Antiarrhythmics (e.g. amiodarone, disopyramide, dofetilide, ibutilide, procainamide, quinidine, hydroquinidine, sotalol).
• Neuroleptics (e.g. phenothiazines, sertindole, sultopride, chlorpromazine, haloperidol, mesoridazine, pimozide, or thioridazine), antidepressive agents.
• Certain antimicrobial agents, including agents of the following classes:
- macrolides (e.g. erythromycin, clarithromycin),
- fluoroquinolones (e.g. moxifloxacin, sparfloxacin),
- imidazole and triazole antifungal agents,
- and also pentamidine and saquinavir.
• Certain non-sedating antihistamines (e.g. terfenadine, astemizole, mizolastine).
• Cisapride, droperidol, domperidone, bepridil, diphemanil, probucol, levomethadyl, methadone, vinca alkaloids, arsenic trioxide.
Have taken a DHA/PQP dose in the previous four weeks
Family history of sudden unexplained death, or personal or family history of predisposing cardiac conditions for arrhythmia/QT prolongation (including congenital long QT syndrome, arrhythmia, QTc interval greater than 450 milliseconds with either Bazett or Fridericia correction).

<u>About</u> 10,000 patients will be included in the study. A subgroup of <u>about</u> 1,000 patients will be assessed more closely. Each patient will be included once.

Several public health facilities in up to 7 HDSS sites within the HDSS in Burkina Faso, Mozambique, Ghana, and Tanzania will be involved.</td></tr>
<tr><td>Study Flow Charts</td><td>Reported below.</td></tr>
<tr><td>Evaluation criteria</td><td>

Clinical safety will be assessed through the analysis of adverse events (frequency, intensity, action taken, outcome) captured during their follow up contacts on Day 5 (±2 days) after starting the treatment with Eurartesim® as well as those identified in the referring hospitals or through adverse events spontaneously reported by the patient detected at the health facility within 28 days after the first medication intake.
Special effort will be put on recording and assuring adequate follow-up to serious and/or severe adverse events and to the adverse events of special interest (see specific section).
A nested subset population of <u>about 1,000</u> patients will have more intensive blood and ECG monitoring. Haematology (Hb and full blood counts (RBC, WBC and differential count)) and standard biochemistry (BUN, Creat, ALT/AST, Bilirubin, electrolytes (K^+ and Cl^-)), as well as ECG will be evaluated at baseline (Day 1 before drug administration), Day 3 (before the last drug administration (not for hematology and blood chemistry) and 3-4 hours after the last drug administration), and Day 7. In case of clinically significant abnormalities, the examination will be repeated. The occurrence of adverse events will be recorded during these visits. In predefined centres having the capability to store plasma samples, all the 1,000 subset will also perform a blood drawing for PQ plasma
</td></tr>
</table>

<table>
<tr><td></td><td>concentration at Day 1 (before drug administration), twice at Day 3 (before and 3-4 after the last drug administration) as well as on Day 7.
• The relationship between the occurrence of adverse events and the administration of concomitant medications will be evaluated in the subset of 1,000 patients.
• Compliance to study treatment will be assessed on the whole population by asking to the patient if he/she adhered with the treatment prescription on the 5 ± 2 days contact. In the 1,000 patient subset, compliance will be evaluated counting the number of tablets dispensed but not taken.</td></tr>
<tr><td>AEs of special interest (AESIs)</td><td>In case of an AESI confirmed by the study doctor, the sponsor shall be informed within 24 hours, even if the event does not satisfy any condition of seriousness. Notification will occur through the use of an ad hoc AESI form. AESIs can be related to:

• Cardio-toxicity i.e. prolonged QT
• Neurotoxicity/seizures
• Cutaneous reactions/phototoxicity

The HDSS study team, as well as the relevant referral health facilities, should be trained to take particular notice of symptoms/signs suggestive of AESIs in this study:
-clinical signs of possible cardio-toxicity/prolonged QT such as:

• Palpitations
• Seizures
• Pounding/pain in the chest area
• Fainting/syncope.

- clinical signs of possible neurotoxicity/seizures such as:

• Seizures
• Dizziness
• Pins and needles sensations
• Visions disturbance
• Difficulties coordination
• Tinnitus

-clinical signs of possible Cutaneous reactions/phototoxicity such as:

• Urticaria
• Angioedema
• Skin lesions
• Itching pruritus
• Discoloration
• Dermatitis

In all these cases, patients shall be directed to the health facility for evaluation and recording of all relevant information. If an AESI is confirmed, the PI / co-PI should be contacted.

In the case of clinical signs of possible cardio-toxicity, an ECG will be performed as soon as the patient arrives at the health facility to look for the following</td></tr>
</table>

	modifications:<br><br>• QTcF >500ms<br>• Increase of QTcF from baseline >60 ms<br>• T wave liability, or T wave morphologic changes during therapy<br><br>The patient will be asked about the time elapsed between Eurartesim® administration and food intake.<br><br>The study team should be aware of the fact that, although drug-induced prolongation of the QTc interval is usually asymptomatic, signs of the following clinical events could be potentially associated with a QTc prolongation:<br><br>• Torsade de pointes<br>• Sudden death<br>• Ventricular tachycardia<br>• Ventricular fibrillation and flutter<br>• Syncope<br>• Seizure.
**Sample size**	According to the cases registered in the previous years in the area where the study is performed, it should be possible to include approximately 10,000 cases of malaria confirmed by a parasitological diagnosis or diagnosed according to WHO criteria. This number of cases will allow identification of "rare" adverse events (incidence: 1 case/3,000 patients) with a 95% probability.<br><br>The sample size computation for the nested study (~1,000 patients) has been also based on feasibility considerations. The focus of this subset study will be on the combination of AESIs related to cardio-toxicity/prolonged QT (see above). In this subset, there will be a probability of 0.95 (0.865) of observing at least one cardiac AESI assuming that the true incidence of this event is 0.003 (0.002). For such a sample size, if no events have been observed, the upper limit of the 95% confidence interval for the probability of such event is 0.003.
**Statistical analysis**	The statistical analyses will be performed using the software package STATA.<br><br>A detailed statistical analysis plan will be generated within three months of study beginning. This plan may be revised during the course of the study in order to take into account the protocol amendments, if any, and to address unexpected problems encountered during the conduct of the study, which could affect the planned analyses. A final statistical analysis plan shall be produced before the database lock.<br><br>**Analysis in the whole sample and in the Subset population**<br>The statistical analysis will be mainly descriptive. The estimates of the incidence of adverse events will be based on crude rates. All estimates will be complemented with appropriate 95% confidence intervals.<br><br>The adverse events shall be regularly coded using MedDRA before database lock.<br><br>All SAEs and AESIs will be reviewed centrally by an independent safety monitoring panel.<br><br>Incidences will be computed on the following classes of AEs, overall and by SOC/PT:

	▪ All AEs<br>▪ Serious AEs<br>▪ Serious AEs related to Eurartesim® treatment<br>▪ AEs of special interest (AESIs)<br>▪ AEs which caused early discontinuation of Eurartesim®<br>▪ AEs related to Eurartesim® treatment<br><br>Specific analyses will be carried out on the AESIs. Correlation analyses will be carried out in order to verify if their occurrence is more frequent with the previous/concomitant use of other drugs (specific classes to be determined in the statistical analysis plan), including antimalarials, in presence of co-morbidity (specific classes to be determined in the statistical analysis plan), or in presence of parasitaemia.<br><br>Compliance will be computed in the whole population and in the subset of 1,000 patients, calculating for each patient the ratio between the number of tablets taken and the number of tablets that the patient should have taken. Two groups of patients shall be described: compliant = 100% treatment compliance and non-compliant = other cases. The safety profile of compliant vs non-compliant patients will be compared.<br><br>**Additional analysis in the sub-set population**<br><br>QTc data will be recorded and analysed according to the method described in the ICH guideline E14. The impact of all recorded covariates on QTc prolongation will be investigated through analysis of covariance techniques.<br><br>For the AESIs possibly related to cardio-toxicity, a correlation analysis with QTc values recorded at the visit of Day 3 (both before and 3-4 hour after treatment administration) will be carried out. Occurrence of these events will also be studied with respect to distance from food intake.<br><br>Laboratory values will be analysed after normalization. Shift tables from normal to abnormal and vice-versa will be generated.<br><br>All the analyses previously described will be also performed by class of age and by type of Plasmodium.
**Ethical issues**	The protocol will be conducted in compliance with the directives in Ghana, Tanzania, Burkina Faso and Mozambique, in particular concerning the submission to the Ethics Committees and the protection of personal data.
**Dates**	Start of patient enrolment will occur after approval by the regulatory authorities in each country approximately QTR 3, 201**3**. Termination of enrolment is estimated to be within two years after the study start and in any case until 10,000 **treated** malaria cases are enrolled.

STUDY FLOW CHART: WHOLE SAMPLE

STUDY EXAMINATION	Day 1/**V1** Inclusion visit (in the health centre)	Day 5 (± 2 days)/**V2** **(Contact by the CHA)**	**Unforeseen Visit**	**End of 28 day follow-up**
Demographic data, sex and weight	X			
Informed consent and recruitment criteria	X			
Physical examination / Malaria symptoms / Other symptoms	X		x	
Rapid Diagnostic Test/microscopy to confirm diagnosis (if feasible)	X			
Thick blood smear collection for further evaluation	X		x	
Previous medications (last month)	X			
Medical history	X			
Prescription of Eurartesim®	X			
AE recording	X	x#	x	X
Concomitant medications	X	X	x	

In the case of clinical signs of possible cardio-toxicity, an ECG will be performed as soon as the patient arrives at the health centre.

STUDY FLOW CHART: NESTED STUDY

STUDY EXAMINATION	Day 1/**V1** Inclusion visit (in the health centre)	Day 3 and Day 7 **V2 and V3** (at the hospital)	**Unforeseen Visit**	**End of 28 day follow-up**
Demographic data, sex and weight	X			
Informed consent and recruitment criteria	X			
Physical examination / Malaria symptoms / Other symptoms	X		X	
Rapid Diagnostic Test/Thick blood smear for Microscopic confirmation of diagnosis	X		X	
Previous medications (last month)	X			
Medical history	X			
Hb, RBC, WBC (Differential Count), BUN, Creat, AST/ALT, Bilirubine, K^+ and Cl	X	X	x(**)	
Prescription of Eurartesim®	X			
AE recording	X	X	X	X
ECG recording	x (*)	x#	x (**)	
Concomitant medications	X	X	X	
Plasma sample collection for PQ concentration	X	x#	X	

(*) Triplicate ECG will be recorded, without causing delay in the treatment of malaria
(**) If clinically significant abnormalities are observed at Day 7
On Day 3, ECG recording and plasma sample for PQ will take place twice, i.e. before and 3-4 hours after Eurartesim® administration. The ECG recorded after drug intake will be collected in triplicate.

2. LIST OF ABBREVIATIONS

ACT	Artemisinin-based Combination Therapy
AE	Adverse Event
AESI	Adverse Event of Special Interest
AS	Artesunate
BF	Burkina Faso
CHA	Community Health Agent
CRF	Case Report Form
DHA	Dihydroartemisinin
DOT	Directly Observed Treatment
ECG	Electrocardiogram
HCP	Health Care Provider (of the HDSS centre)
HDSS	Health **a**nd Demographic Surveillance S**ystem**
GEP	Good Epidemiological Practices
GH	Ghana
HA	Health Agent (of the HDSS centre)
INESS	The INDEPTH Effectiveness and Safety Studies
MA	Marketing Authorisation
MMV	Medicines for Malaria Venture
MZ	Mozambique
NMCP	National Malaria Control Program
PHP	Public Health Pharmacy
PI	Principal Investigator
PQP	Piperaquine phosphate
RDT	Rapid Diagnostic Test
SAE	Serious Adverse Event
SmPC	Summary of Products Characteristics
TZ	Tanzania
WHO	World Health Organisation

3. INTRODUCTION AND RATIONALE

3.1 Summary of current situation

In response to the break out and the spreading of classical drug-resistant plasmodium strains, the WHO recommends the use of artemisinin-based combination therapies (ACTs) in the treatment of uncomplicated malaria episodes[1].

New ACTs are entering the African market and will be used by the public health care system. Until now, these ACTs were prescribed to a limited number of patients and most of the time within strictly regulated clinical studies with patients meeting restrictive selection criteria. The collection of safety data and the risk evaluation based upon observational data are critical in order to evaluate the risk/benefit profile of a product through its life cycle and allow providing information on the best use of medicines. In addition, the impact of the introduction of a particular ACT in the public health care system on the evolution of its efficacy and on the malaria morbidity and mortality is unknown and is worth being investigated.

3.2 Eurartesim®

Eurartesim® is a fixed-dose combination product composed of dihydroartemisinin (DHA) and piperaquine phosphate (PQP) for the oral treatment of uncomplicated *Plasmodium falciparum* malaria. Eurartesim® contains DHA, to which all malaria strains are susceptible and to which drug resistance has not been reported. The long-term efficacy of Eurartesim® is secured by the presence of PQP. PQP is a second anti-malarial compound with a different mechanism of action and much longer half-life. Eurartesim® appears to offer significant benefits over existing licensed malaria treatments and is in line with current WHO treatment policy recommendations.
Two pivotal trials[2,3] on the efficacy of Eurartesim® on treatment of uncomplicated *Plasmodium falciparum* malaria have been conducted in Africa and Asia, showing a high rate (>95 %) of treatment success. The Eurartesim® registration dossier has been submitted to EMA authorities and it got a centralized marketing authorization.
Evaluating possible drug-related side effects in patients with malaria and treated in the endemic countries can be difficult. There is considerable overlap between treatment-related symptoms and signs, and those of malaria (such as fever, anaemia) and concomitant illnesses (such as viral infections and parasitic infestations). Other reported AEs have been headache, vomiting, abdominal pain, asthenia, and cough.
The potential for Eurartesim® to prolong the QTc interval was investigated in parallel groups of healthy volunteers co-administered with high (~1,000 Kcal) or low (~400 Kcal) fat/calorie meals or in fasting conditions. Compared to placebo, the maximum mean increases in QTcF following Eurartesim® administration were 45.2, 35.5 and 21.0 msec, respectively, on day 3. Such QTcF prolongation observed under fasting conditions lasted between 4 and 11 hours after last dosing on day 3; afterwards, the mean QTcF prolongation compared to placebo decreased to 11.8 msec at 24 hours and to 7.5 msec at 48 hours. No healthy subject dosed in fasting conditions showed a QTcF greater than 480 msec, or an increase over baseline greater than 60 msec. The number of subjects overpassing these thresholds were 3 and 10 out of 64, respectively, for the low fat/calorie condition and 3 and 14 out of 40 for the high fat/calorie

condition. No subject had a QTcF value greater than 500 msec in any of the dosing conditions.
More details about Eurartesim® can be found in the SmPC of the product.

DHA is very rapidly absorbed, the Tmax being about 1-2 hrs after single and multiple dosing. DHA bioavailability resulted higher in malaria patients than in healthy volunteers, possibly because malaria *per se* has an effect on DHA disposition, which may reflect malaria-associated impairment of hepatic function, causing an increase in DHA bioavailability (reduction of hepatic first pass effect) without affecting its apparent elimination half-life, which is absorption rate limited.
Piperaquine (PQ), a highly lipophilic compound, is slowly absorbed. In humans, it has a Tmax of about 4-5 hours after single and repeated dose. Due to its slow elimination, PQ accumulates in plasma with an accumulation factor of approximately 3 after the scheduled multiple dose treatment of three days. In healthy volunteers, PQ exposure is increased approximately 3-fold when administered with a high fat/high calorie meal. This effect is of clinical relevance because PQ is responsible for the effect of Eurartesim® on QT interval. Accordingly, Eurartesim® should be administered with water far away from meals.
DHA is principally converted to α-DHA-β-glucuronide (α-DHA-G). Studies in human liver microsomes showed that DHA was metabolised by the UDP-glucuronosyltransferase (UGT1A9 and UGT2B7) to α-DHA-G with no cytochrome P450-mediated metabolism. *In vitro* drug-drug interaction studies revealed that DHA is an inhibitor of CYP1A2, therefore there is the potential for DHA to increase plasma concentrations of CYP1A2 substrates.
The metabolism of PQ in humans has not been studied *in vivo*. *In vitro* metabolism studies demonstrated that PQ is poorly metabolized by human hepatocytes (about 85% of PQ remained after 2 hours incubation at 37°C). PQ was mainly metabolised by CYP3A4 and to a lesser extent by CYP2C9 and CYP2C19. PQ was found to be an inhibitor of CYP3A4 (also in a time-dependent way) and to a lesser extent of CYP2C19, while it stimulated the activity of CYP2E1. As a consequence, there is the potential for increasing plasma concentrations of CYP3A4 substrates and also for the increase of PQ plasma concentrations when Eurartesim® is concomitantly administered with CYP3A4 substrates and CYP3A4 inhibitors, respectively.
The elimination half-life of DHA is approximately 1 hour. Data regarding DHA excretion in humans are scarce. However, it is reported in the literature that the excretion of unchanged drug in human urine and faeces is negligible for artemisinin derivatives.
The elimination half-life of PQ is around 22 days for adult patients and around 20 days for paediatric patients. *In vitro* metabolism studies demonstrated that PQ is poorly metabolized by human hepatocytes and its major metabolites were a carboxyl acid cleavage product and a mono-N-oxidated product.
Animal studies showed that radiolabelled PQ is excreted by the biliary route, while urinary excretion is negligible.

3.3 Malaria in Burkina Faso, Mozambique, Ghana and Tanzania

Ghana

Malaria is the leading cause of morbidity and mortality reported in health facilities in the Northern areas where N**a**vrongo HDSS is located. **Malaria transmission occurs during most months of the year but there is a distinct seasonal pattern with the peak of**

transmission coinciding with the period of the major rains (May – October) and the dry season (November – April) seeing very low rates of malaria infection. Transmission has been estimated to be 418 infective bites per person per year[4-6]. The main malaria vectors are *A. gambiaes.l* and *A. funestus* constituting about 94.3% of the vector population. The predominant parasite species is *P. falciparum* (accounting for more than 90% of all infections). *P. malariae* is seen in a small proportion of infections (less than 5%) and some mixed infections of *P. falciparum* and *P. malariae*. *P. ovale* is rarely seen and no *vivax* is seen[5,6]. The incidence of clinical malaria also shows some seasonality with an incidence density of infection varying from five cases per person- year in the dry season to over seven cases per person-year in the wet season[7]. In a just concluded study of malaria morbidity and mortality in a birth cohort of children in the district (n=2200), 38.9% of all admissions in this cohort was due to malaria with malaria accounting for 50.9% of all hospital visits made by cohort members (unpublished data).

In the middle belt of Ghana where the Kintampo HDSS is located, The parasite prevalence in children <10 years of age is >50% all year round and the incidence of clinical malaria among children less than 5 years is about 7 episodes per child per year[8]. The main vectors for transmission of malaria are *Anopheles gambiae* and *A. funestus* and the transmission is perennial but peaks between April and October. The annual entomological inoculation rate was 269 infective bites per person per year in 2005[9]. Malaria is diagnosed by clinical suspicion and confirmed with rapid diagnostic test or microscopy. The first line treatment for malaria is artesunate-amodiaquine or arthemether-lumefantrine for uncomplicated malaria and quinine for complicated malaria. Chemical shops are the first point of call during a child's febrile illness and use of traditional medicine and left over drugs at home is also common in the study area[10].

Tanzania

Overall malaria is on decline in Tanzania[11]. However, it is still a very important public health problem in some areas including INESS study sites. Community based cross sectional studies that were conducted of late suggest population level parasite prevalence of about 14% in Rufiji and 3% in Kilombero-Ulanga health and demographic surveillance system (HDSS) sites (*Results from INESS study*). The infection is largely caused by *Plasmodium falciparum* parasites that are transmitted by *A. gambiae*, *A. arabiensis* and *A. funestus mosquitoes*[12]. Malaria transmission in these two HDSS sites is intense and perennial. However, the disease becomes most prevalent from February to September, during and immediately after long rains. The main control strategies in existence are case management with artemether-lumefantrine and use of insecticide treated nets (ITNs).

Burkina Faso

Burkina Faso a landlocked country is located in the heart of Africa. It is composed into 13 administratives regions that includes 45 provinces. The Kossi province belongs to Mouhoun region and borders the Republic of Mali. Nouna is located 297 km Northwest from Ouagadougou, the capital of the country. The Kossi province corresponds geographically to the Nouna Health District area, which has a size of 7464,44 km^2 and a population of about 320,000 in 2009[13]. The HDSS operating since 1992, entirely belongs to this area. The area is a dry orchard savanah, inhabited and mainly by subsistence farmers and cattle keepers of different ethnic groups. The short rainy season usually lasts from june to October[14]. The dry

season includes 2 types: a dry, cold and dusty period (November to February) and a dry and very hot period (March to May)
The annual rainfall in this area varies between 500-1000 mm. Throughout the year, the minimum and maximum daily ambient temperatures are approximately 20°C and 40°C. Malaria is defined as holoendemic in the area with most transmission occurring during or shortly after the rainy season and 99% being attributed to *P. falciparum*. July to December is considered as the high malaria transmission season, while the period January to June is considered as a low transmission season. Access to malaria prevention has remained rather limited until very recently with only one quarter of households possessing ITNs in 2007(15), but this proportion is increased to 59% in 2010(16). Access to quality malaria treatment was and is difficult in this rural study area(17). Only 14.6% of malaria cases are diagnosed by microscopy (Unpublished data). Only 15% of children with malaria having received early ACT in 2010(16).

Mozambique
The Manhiça Health Research Center (CISM – Centro de Investigação em Saúde de Manhiça), is located in Manhiça district, Maputo province, southern Mozambique. CISM maintains in its study area a demographic surveillance system since 1996 that covers 84.000 inhabitants, of which 44% are younger than 15 years of age(18). The census and household information are updated every six months and all houses are geopositioned. It also runs a morbidity surveillance system at the Manhiça District Hospital (HDM) and 5 health centers in the area. A standardized questionnaire is filled out for all visits to the outpatient clinic and admissions to hospital of children younger than 15 years, where demographic information, symptoms and signs of the current illness, results, diagnosis, treatment and outcome are recorded. In all children with fever (axillary temperature ≥37.5ºC) or history of fever in the previous 24 hours a finger prick is performed to measure hematocrit and prepare blood smears to determine parasitemia. Only children with a positive blood smear receive antimalarials, currently artemether plus lumefantrine (AL) for uncomplicated malaria. The study area covers around 500 km^2 and includes semi-rural and rural areas, where houses are typically made of cane walls with thatched or corrugated iron roofs. The population is of the Shangana-Ronga ethnic group and is mainly composed of subsistance farmers, petty traders and workers in a nearby sugar cane factory.

There are two distinct seasons: a warm and rainy season from November to April and a cool dry season the rest of the year. The transmission of malaria in the area is stable with seasonality, with *P. falciparum* being responsible for the majority of the cases and *A. funestus* being the main vector. Transmission, that used to be of moderate intensity, with an entomological inoculation rate of 38 infective bites/person/year in 2002(19), has decreased in the last few years. Malaria preventive measures in place in Manhiça include diagnosis of malaria cases with blood smears or rapid diagnostic tests, treatment of confirmed cases with AL, the free distribution of LLINs to pregnant women through antenatal clinics, indoor residual spraying (IRS) with DDT and intermittent preventive treatment in pregnant women (IPTp) with three doses of sulfadoxine-pyrimethamine as indicated and provided by the NMCP.

In 2010 minimum community-based incidence rates of outpatient malaria cases per 1,000 child years at risk detected through the morbidity surveillance system ranged from 39-220 in

infants, 110-764 in children aged 1 to <5 years and 64-274 in children aged 5 to 15 years depending on the area. Malaria accounted for 7-34% of all outpatient visits in children younger than 15 years depending on the area. In community cross-sectional surveys in 2010 and 2011 the prevalence of parasitaemia was <15% for all age groups and areas.

3.4 Project rationale

The project will take place within up to 7 HDSS centres located in four countries and it has a double interest. The first interest is to collect safety data in real conditions of use on a medicine the safety profile of which is well known only through classical clinical studies.
The second interest is to develop new methods for actively performing pharmacovigilance, which will subsequently be used for other anti-malarial drugs, and drugs in other therapeutical classes in sub-Saharan Africa.

This program is being implemented in close collaboration with the NMCP of each of the four countries and the Pharmacovigilance Department of the Sponsor or his/her Designee. The collected data will be regularly submitted to an independent committee in charge of safety monitoring, as well as to the Health Authorities and will be communicated to the WHO collaborating pharmacovigilance centre in Uppsala.

3.5 Proposed Sites participating to the study

1) Dodowa HDSS, Ghana
2) Kintampo HDSS, Ghana
3) Navrongo HDSS, Ghana
4) N**o**una HDSS Burkina Faso
5) Rufiji HDSS Tanzania
6) Manhiça HDSS site, Mozambique
7) **Nanoro HDSS Burkina Faso**

Other sites **might** be brought on board by the sponsor in case of need to execute the study optimally and achieve the required sample size.

4. STUDY OBJECTIVES

4.1 Primary objective

The primary objective is the evaluation of the clinical safety of Eurartesim® in standard conditions of use (unsupervised treatment intake), on a population of approximately 10,000 patients with signs and symptoms of uncomplicated malaria confirmed by a parasitological diagnosis or, when this test is not available, by WHO diagnostic criteria.

This sample will allow the detection of rare adverse events (i.e. with an incidence of 1 case per 3,000 patients).

4.2 Secondary objectives

- 4.2.1 Main secondary

- Intensive assessment of a nested subset of **about 1,000** patients to evaluate the effect of the administration of Eurartesim® on blood biochemistry, full blood count and QTc intervals.
- QTc interval and piperaquine concentration relationship will also be investigated in all the **nested 1,000** patients.

.

- 4.2.2 Other secondaries
  - Description of the prevalence of *Plasmodia* species (*falciparum, vivax, ovale, malariae*) as confirmed by blood smear results in the nested subset of 1,000 patients and retrospective comparison of the tolerability profiles between the two sub-populations of patients infected with *Plasmodium falciparum* and patients infected with other *Plasmodia.*

  - Assessment of the relationship between the occurrence of adverse events and the administration of concomitant medications will be made in the subset of 1,000 patients.

5. DESCRIPTION OF THE STUDY

5.1 Description of the study design

This is an observational, non-comparative, longitudinal study, conducted on approximately 10,000 patients with signs and symptoms of uncomplicated malaria suspected or confirmed by a parasitological diagnosis or, when this test is not available, by WHO diagnostic criteria.

A subset of **about 1,000** patients will be assessed more closely for QT interval prolongation and laboratory tests. In this subset, the presence of *Plasmodia* of any species will be confirmed by microscopy. **Plasma samples from all the 1000 patients will also be taken for evaluation of** PQ concentration at different timepoints. From such blood drawings and before centrifugation, three drops of whole blood will be spotted on filter papers. These filter papers will be utilized to determine whole blood piperaquine concentration with the Dry Blood Spot methodology (if a validated analytical method for such determination will be settled up at the time of the study course).

The study will take place in several public health facilities in up to **7** HDSS centres (Health and Demographic Surveillance Sites) in Burkina Faso (**2**), Mozambique (1), Ghana (3), and Tanzania (1), where Eurartesim® will be used as first-line treatment of uncomplicated malaria episodes. The patients will be admitted to the entry visit following the usual procedures of the HDSS centre. The subset of **about 1,000** patients will be constituted by patients who agree to participate in the closer monitoring conditions.

In case of treatment inefficacy or safety concerns, medicines recommended by the NMCPs of each country will be prescribed as rescue treatments.

All patients visiting one of the health facilities referring to the HDSS centres and for whom a diagnosis of uncomplicated malaria (according to the WHO criteria) is suspected or confirmed by a parasitological diagnosis will be enrolled in the study after their informed consent (a parent's or guardian's consent for**m/signature will be required for** children **as per each country regulation**) has been obtained. For **teenagers (age depending on country regulations**), an assent to participate in the study will be also sought if required by the National guidelines. A thick blood smear will be prepared for microscopic diagnosis. **For the participants consented into the main group, e**urartesim® tablets will be prescribed to the patients (or to the parents/guardians if the patients are children) meeting the protocol inclusion criteria before the results of the thick blood smear are known **if malaria has already been confirmed from RDT and/or clinical examination as per country regulations.**

The patients will be contacted at Day 5 (± 2 days) by a community health agent (CHA) of the HDSS, in order to capture recovery status and all the experienced adverse events. A visit by the community health agent will be scheduled on all the cases in which the information collected during the telephone contact should be considered incomplete or unreliable ones. Special procedures will be followed in case of serious and/or severe adverse events and events classified of special interest (see specific section).

The subset of 1,000 patients will be intensively followed-up. These patients will have haematology (Hb and full blood counts (RBC, WBC and WBC differential count)) and standard biochemistry (BUN, Creat, ALT/AST, Bilirubine, electrolytes (K+ and Cl^-)) undertaken at Day 1 (before drug administration), Day 3 (3-4 hours after the last dose of treatment), and Day 7.

Each blood sampling for both haematology and biochemistry requires about 2mL of venous whole blood. This makes up for a total amount of about 6mL of venous whole blood to be taken from each participant in the nested group for the entire study period.

If the results are abnormal, the blood examination will be repeated until normalization. Triplicate ECG and blood sampling for PQ concentration assessments (in all the 1,000 patient subgroup) will be performed on Day 1 (before drug administration); single ECG and blood sampling for PQ concentration (in all the 1,000 patient subgroup) will be repeated on Day 3, before the last dose of Eurartesim® and 3 to 4 hours after the last drug administration (in this latter case ECG will be performed in triplicate), as well as on Day 7; safety information will be collected at all these visits. **Each PK sample will be 2 mL of whole blood which makes a total of 8 mls for the entire study period unless clinically indicated.**

If the QTcF (QT corrected by Fridericia's formula) value assessed on Day 3 before last dose is above 500 ms, Eurartesim® should be withheld until QTcF returns below 480 ms within 6 hours. Thereafter, the Eurartesim® cycle may be completed under frequent QTc monitoring based on medical judgment. If the QTcF should not return below 480 ms within 6 hours, another antimalarial therapy should be considered.

The occurrence of any adverse events will be solicited from the subset of **about 1,000** patients on Days 3 and 7 following administration of Eurartesim® as well as in any additional visits.

All patients in the study with a cardiac event of special interest (see section 8.5) will have an ECG performed.

Patients will be asked to report to the health facility or to the HDSS if any adverse event occurs after Day 5 (± 2 days) contact and within 28 days after the start of Eurartesim® intake.

Female patients will be encouraged to communicate to the study team if they get pregnant within a period of two months after the start of the Eurartesim® treatment. In these cases, information on the evolution of the pregnancy will be collected at 3, 6, 9 months and after the delivery (6 and 14 weeks). Information on the drugs taken during the pregnancy as well as AEs/SAEs/AESIs and the health status of the newborn/s will be collected.

5.2 Study duration

The study will last approximately two years.

5.3 Drug prescription and dosing

The patients will be instructed to take Eurartesim® (DHA/PQP) with a dose regimen of one administration every 24 hours over a period of three days, i.e. at Day 1, then after 24 hours (Day 2) and after 48 hours (Day 3) from the first administration.

The dose will be based on body weight **as depicted in the table below.** Two strengths of Eurartesim® will be provided to facilitate the dosing in children and adults: 20/160mg and 40/320mg of DHA and PQP respectively.

**Body weight (kg)**	**Daily dose (mg)**	**Number of tablets per dose**	
		20/160mg DHA/PQ	40/320mg DHA/PQ
5 to <7	10 mg DHA and 80 mg PQP	**½ tablet**	
7 to <13	20 mg DHA and 160 mg PQP	**1 tablet**	
13 to < 24	40 mg DHA and 320 mg PQP		**1 tablet**
24 to < 36	80 mg DHA and 640 mg PQP		**2 tablets**
36 to < 75	120 mg DHA and 960 mg PQP		**3 tablets**
75 to < 100	160 mg DHA and 1280 mg PQP		**4 tablets**
> 100	There are no data on which to base a dose recommendation		

The patients will be instructed to take Eurartesim® with water, at least three hours before or three hours after food intake (i.e. three hours after the previous food intake with no food intake for the following three hours after Eurartesim® administration).

To facilitate drug administration in small children, tablets will be crushed on a spoon and given with water. If vomiting occurs within 30 min from drug administration, dose will be re-administered. If vomiting occurs within 30 to 60 min, half a dose have to be re-administered. Re-dosing should not be attempted more than once. The first study drug administration will be supervised at the site in all the patients, instruction on the second and the third drug administration will be given to patients.

In the nested 1000 patient group, the second administration will be given preferably at the study site or at the patient's home by a trained CHA. If the dose is administered at home, a specific diary will be provided to the patient in which the CHA have to fill in the second drug administration data. This diary will be maintained by the patient and will be returned to the site when the third administration will be provided (at the site).
Eurartesim® will be provided free of charge by Sigma-Tau ifr S.p.A.

5.4 Evaluation criteria

Primary evaluation criterion: The clinical safety of Eurartesim® will be evaluated through the analysis of the adverse events captured by the health agent that will contact each patient at Day 5 (±2 days) after starting of Eurartesim® treatment. Any AE referred (frequency, intensity, action taken, outcome) will be recorded on the specific section of the patient chart as well as those identified in the referring hospitals or through adverse events spontaneously reported by the patient detected at the health facility within 28 days after the first medication intake. Special attention will be given to the serious adverse events (SAE) and/or adverse events (AE) classified as severe and/or adverse events of special interest (AESI) (see section 8.5).

- Main secondary evaluation criteria: The cardiac and biological safety of Eurartesim® will be intensively evaluated in the nested subset of **about 1,000** patients to evaluate the effect of the administration of Eurartesim® on blood biochemistry (BUN, Creat, ALT/AST, Bilirubin, electrolytes (K+ and Cl^-)), full blood count (Hb, RBC, WBC and differential count)) as well as ECG parameters. In case of clinically significant abnormalities, the examination will be repeated. The occurrence of adverse events will be recorded during these visits. Concentrations of PQ will be evaluated in a subgroup of about 200 patients at baseline (Day 1 before drug administration), Day 3 (before the last drug administration and 3-4 hours after the last drug administration), and Day 7. QTc interval and piperaquine concentration relationship will also be investigated.

Other secondary evaluation criteria

- The results of the thick blood smears systematically prepared at Day 1 in the subset of **about 1,000** patients will allow a retrospective categorization of patients in the two subsets of those confirmed to be infected with *Plasmodium falciparum* malaria and those infected with other *Plasmodia*. A comparison of the tolerability profiles between these sub-populations will be carried-out.

- The relationship between the occurrence of adverse events and the administration of concomitant medications will be also evaluated in the subset population of **about 1,000** patients.

6. STUDY POPULATION AND PATIENT SELECTION

6.1 Sample size

About ten thousand patients with suspected malaria diagnosed by WHO criteria and possibly by a RDT/microscopy will be enrolled and a subset of **about 1,000** patients with a microscopy diagnosis of *Plasmodia* (any species) will be studied more intensively

6.2 Selection criteria

6.2.1 Inclusion criteria

- Uncomplicated malaria (*Plasmodia* of any species) diagnosed as per national policies and in line with WHO recommendations (a history of fever in the previous 24 h and/or the presence of anaemia, for which pallor of the palms appears to be the most reliable sign in young children). Confirmation of malaria by a parasitological diagnosis with RDT is encouraged but its absence does not prevent patients from being enrolled.
- Age ≥ 6 months and weight ≥ 5 kg.
- Capability of taking an oral medication.
- Ability and willingness to participate based on signed informed consent (a parent or a guardian has to sign for children below 18 years old), or on verbal consent given in front of a witness signing the informed consent, and access to health facility. The patient is to comply with all scheduled follow-up visits.

6.2.2 Exclusion criteria

- Known allergy to artemisinin or to piperaquine.
- Known pregnancy.
- Lactating women should be excluded if other anti-malarial treatments are available
- Complicated malaria.
- Taking medicinal products that are known to prolong the QTc interval. These include (but are not limited to):
  - Antiarrhythmics (e.g. amiodarone, disopyramide, dofetilide, ibutilide, procainamide, quinidine, hydroquinidine, sotalol).
  - Neuroleptics (e.g. phenothiazines, sertindole, sultopride, chlorpromazine, haloperidol, mesoridazine, pimozide, or thioridazine), antidepressive agents.
  - Certain antimicrobial agents, including agents of the following classes:
    - macrolides (e.g. erythromycin, clarithromycin),
    - fluoroquinolones (e.g. moxifloxacin, sparfloxacin),
    - imidazole and triazole antifungal agents,

- and also pentamidine and saquinavir.
  - Certain non-sedating antihistamines (e.g. terfenadine, astemizole, mizolastine).
  - Cisapride, droperidol, domperidone, bepridil, diphemanil, probucol, levomethadyl, methadone, vinca alkaloids, arsenic trioxide.
- Have taken a DHA/PQP dose in the previous four weeks.
- Family history of sudden unexplained death, or personal or family history of predisposing cardiac conditions for arrhythmia/QT prolongation (including congenital long QT syndrome, arrhythmia, QTc interval greater than 450 milliseconds with either Bazett or Fridericia correction).

6.3 Enrolment procedure

6.3.1 Investigational Sites

The Study will be conducted at selected health facilities in the following HDSS, Manhica, Rufiji, Dodowa, Kintampo, Navrongo, Nouna **and Nanoro**.

6.3.2 Patient selection

At least 10,000 **treated** malaria cases will be enrolled. A subset of about 1,000 patients will be assessed more closely. Each patient will be included once.

A patient will be included in the study in case he/she meets the selection criteria of the protocol and agrees to participate. In particular, in order to enter the study, the patient should accept that one drop of blood is taken for the preparation of a thick blood smear, that the community health agent contact him/her at home, that the data collected during those visits are registered and analyzed and finally that, in case of an SAE and/or an AE classified as severe and/or an AESI (see section 8.5) should occur he/she has to contact immediately the community health agent or the HDSS, directly.

The subset of **about 1,000** patients will need to accept to be strictly monitored at the health facility or HDSS, where a capillary blood sample is taken in order to prepare a thick blood smear, a venous blood sample is collected for chemistry, full blood count and PQ concentration assessment (in all the 1000 patient subgroup), and a triplicate ECG recording is performed before the start of the treatment. Furthermore, he/she has to agree to be visited again on day 3 and day 7 after start of the treatment, to run all the evaluations foreseen by the study protocol.

6.3.3 Patient numbering

Each patient will be identified at the study site by a 7 digits number, referred to as patient ID number. The first digit is the HDSS number, the following two digits are identifying the recruiting health facility and the last four digits will identify the patient enrolment number. Each number will be unique and, in case a patient discontinues the study before the end of the follow-up period, his/her number will not be assigned to another patient.

A second number (nested number) will be assigned to the patients of the nested subset of **about 1,000** patients. This number will be of 4 digits and a chronological recruitment order will be followed in each site.

7. STUDY PROCEDURE AND DATA COLLECTION

7.1 Visit planning

The inclusion in the study will be performed in the HDSS centre or in one of the associated health facilities, at Day 1 during the consultation.

The first follow-up contact with the patient **enrolled in the main group** will be performed at Day 5 (±2 days), always after administration of the last Eurartesim® dose, by the village community health agent.

In addition, the patients will be requested, in the event of any unusual significant symptom experienced during the 28 days following the treatment prescription, to visit their village community health agent (CHA) or contact directly the health agent who followed him/her during the study.

In case of occurrence of a SAE, an AE classified as severe or an AESI (see section 8.5) or if requiring medical care, the patient will be transferred to the study designated health facility by the village community health agent in order to receive care for his/her symptoms (this will be an unforeseen visit) and to provide more detailed information on the event.

In the subset of **about 1,000** patients that will be monitored more closely, they will go back home with a questionnaire in order to collect information about the food they eat from D1 to D3 and they will be asked to come to the health facility (HF) at D3 for directly observed treatment (DOT) administration of the 3rd dose.

In this subset, patients will have to come to the health facility site for a final visit at D7.

7.2 Collected data

7.2.1 Registry at the HDSS centre

Each patient visiting one of the designated study health facilities within the HDSS, suspected to have uncomplicated or complicated malaria, regardless of him/her being included in the study, will be recorded in a specific registry by the study team member who has examined him/her. The date of consultation, the age and sex of the patient, the village or neighbourhood he/she lives in, the recent history of any anti-malarial treatment intake, the weight, the clinical signs, the diagnosis, the prescribed treatment (dose and duration) will be reported in this document as well as the patient ID number (if recruited in the study) or the possible reason for not participating in the study. In this registry, for the patients included in the study, will be also reported the date on which the patient's chart was provided to the CHA (by the

HF) as well as the date in which the CHA consigned the filled in chart with data referring the follow-up contact at Day 5 (±2 days) at the HF; those dates will be reported for traceability purposes.

7.2.2 Examination and data collection at inclusion

In case the patient meets the protocol selection criteria and agrees to participate in the study, the following procedures will be followed:

- An informed consent form will have to be signed and will be filed with the registry.
- The HF will assign a patient ID number to the patient, visit the patient as usual and record the following data:
  - On **the** patient chart: last name, first name, full address (village/neighbourhood), registration number and patient ID number.
  - On the rest of patient chart: the other information collected during this visit, i.e.:
    - Demographic data of the patient: patient ID number, initials, age, sex, weight and body temperature.
    - Intake of anti-malarial drugs during the 4 weeks before inclusion.
    - Intake of any other treatment including traditional treatments, during the 2 weeks before inclusion.
    - Concomitant treatments and treatments prescribed when visiting the centre.
    - Malaria and associated disease symptoms will also be reported.
    - Confirmation that a thick blood smear identified by the patient's ID number and the date of blood withdrawal has been taken and prepared for later reading.
    - Confirmation that a blood sample for haematology, blood chemistry and PK (where foreseen) has been drawn in the subset of patients more closely evaluated; and that in the same subgroup a triplicate ECG has been recorded. Results of the ECGs local readings and laboratory examinations will be reported as soon as possible on the chart (copy of the results have to be stored at site).
    - Eurartesim® treatment administration time. Dosage will be defined according to the weight of the patient. The patient will be instructed to take the medication with water, between meals as described in section 5.3. In all the 10,000 patients the first dose of the study drug will be directly administered at the facility and time of administration and time of last food consumption will be annotated.

7.2.3 Follow-up visits/contacts and clinical tolerability follow-up questionnaire

At Day 5 (±2 days) (after administration of the last Eurartesim® dose) following the inclusion date, the community health agent (CHA) will contact the patient at home. A further visit at the patient's home will be scheduled by the community health agent in all the cases in which the information collected during the telephone contact should be considered incomplete or unreliable ones.
He/she will then fill-in the second part of the chart (in triplicate) and report:

- His/her own 4 digit identification number (first two digits are the number of the health centre he/she is attached followed by a 2 digit number which will be assigned to him/her when the study starts).
- Date of the visit/contact.
- Clinical condition (recovered status or continuation of the illnesses).
- Compliance with the prescribed Eurartesim® treatment and in case, number of tablets of Eurartesim® not taken; an explanation of the reason why the treatment was not followed should be provided.
- Description of the symptoms experienced by the patient in case of a positive answer to the question: "*Have you noticed any unusual event/symptom since your last visit at the centre?*" Collect date of first manifestation of this/these event(s), symptom(s), intensity, treatment dis-/continuation, and evolution, in the specific section of the patient chart dedicated to the collection of Adverse Events.
- Current treatments, if taken.

In case, during his/her contact, the CHA assess that an occurred event is serious and/or severe, or detects an adverse event of special interest (see section 8.5), he/she should contact the HA and transfer the patient to the health facility in order to be examined. If necessary in the opinion of the HA, he/she should contact the investigators so that they can visit the patient and exactly define the status of the event and the causality. If the adverse event belongs to the cardiovascular category, an ECG will be performed. In addition, a thick blood smear will be prepared (for future analysis). In that event, a specific adverse event report form will be completed by the HA or the investigators.
The patient will receive first care treatment. In case any other treatment is prescribed by a HA staff or the study doctor, it will be reported on the patient chart.

All the patients will be instructed to refer to the CHA or the designated HF in the HDSS should any adverse event occur within the 28 day after the start of the Eurartesim® administration.
In addition the regional hospital will be alerted to check referrals for participation in the study, and there will be a collection on a regular basis of any data related to patients who are treated in regional hospitals who have taken part in the study.

The patients recruited in the 1,000 subset group will be reviewed at the HF on day 3 (last day of administration of Eurartesim® dose). Before the Eurartesim® administration, a blood sample will be collected for PQ concentration evaluation (in all the 1000 subgroup of patients) and a single ECG will be recorded. The last dose of Eurartesim® will be directly administered by the HA to the patient after documentation that the last meal was taken more than 3 hours prior to administration. Three to four hours after the Eurartesim® administration, a blood sample for haematology, blood chemistry and PQ concentration will be collected and a triplicate ECG will be recorded.
The following information will be recorded in the second part of the chart (in triplicate):
- The HA or study team staff will record his/her own 4 digit identification number (number of the health centre he/she is attached followed by a 2 digit number which will be assigned to him/her when the study starts).
- Date of the visit.
- Clinical condition (recovered status or continuation of the illnesses).

- Time of drug administration
- Compliance with the Eurartesim® treatment during the three day treatment.
- Results of the ECGs and laboratory examinations
- Occurrence of Adverse Events.

The patients recruited in the subset will have to come back at the health facility on day 7 to perform a further single ECG, a blood sample drawing for haematology and biochemistry determinations and to collect, if any, Adverse Events occurred.
Again the HA or study team staff will record his/her own 4 digit identification number and record the appropriate information on the patient chart.

All the patients will be instructed to refer to the CHA or the designated HF in the HDSS should any adverse event occur starting from the 7 day visit/contact and the 28 day after the start of the Eurartesim® administration.

Female patients will be encouraged to communicate to the study team if they get pregnant within a period of two months after the start of the Eurartesim® treatment. In these cases, information on the evolution of the pregnancy will be collected at 3, 6, 9 months and after the delivery (6 and 14 weeks). Information on the drugs taken during the pregnancy as well as AEs/SAEs/AESIs and the health status of the newborn/s will be collected and reported in the patient chart.

7.3 Patients who did not receive follow-up visits/contacts

7.3.1 Premature discontinuation before drug intake

In case a participant fulfils all selection criteria, he/she is enrolled in the study and is attributed an enrolment number/participant ID number. If for any reason, the participant cannot be given the study drug and must discontinue participation in the study, the reasons for this will be collected by the CHA or HF staff.
These particular subjects will not be part of the safety and efficacy analysis. Additional subjects will need to be recruited in the study in order to make up for the total of 10,000 (9,000 and 1,000) subjects needed for analysis.

7.3.2 Premature treatment discontinuation

The reasons of any premature treatment discontinuation will be collected by the CHA or HF staff during his/her visits/contacts and in case the reason to stop the treatment is the occurrence of an adverse event, this will be described on the specific section of the patient chart and treated depending on its category (see section 8).

7.3.3 Patient lost to follow-up

The study staff will take all appropriate measures to perform each patient's follow-up visits/contacts. However, some patients might be lost to follow up. This information will be recorded in the patient's chart.
A Patient can be considered as lost to follow up if the patient cannot be contacted either by

phone or home visit on scheduled Day (Day 5±2).

In the subset of 1000 patients, a patient can be considered as lost to follow-up if the patient does not present on Day 7 visit and cannot be reached on phone or home visit.

7.4 Thick blood smear logistics

All thick blood smear slides will be stained and stored for independent confirmation of presence or absence of malaria parasites. They will be examined in the laboratory by qualified personnel and according to the laboratory's Standard Operating Procedures. The results will be transferred for data entry in the form of listings. The blood slides will be performed on Day 1 for all the participants..

7.5 ECG Recording

In the subset of **about 1,000** patients, electrocardiograms for safety purpose will be performed using the internationally recognized 12 leads with a ELI 150 Cardiograph®. Printouts of each ECG will be individually identified with the subject initial and the subject number, the date and the time of recording, initials of the technician/nurse, initials of the HA; at least 5 complexes for each lead. The corresponding source data will consist of the Cardiograph paper printouts.

The ECGs will be read and analyzed by the study doctor for safety purpose. ECGs will be also read by a central cardiac laboratory and used for the assessment of the possible impact of Eurartesim® on cardiac activity.

The following parameters will be obtained at each time point following the manual reading: RR (ms), HR (bpm), PR (ms), QRS (ms), QT (ms).

For each patient and at specific time point, the triplicate values will be averaged in order to obtain one single value per subject and time point. From the averaged parameter values the following corrected QT interval will be derived:

– Fridericia's correction: $\mathrm{QTcF\ (ms)} = \dfrac{\mathrm{QT\ (ms)}}{\mathrm{RR\ (s)}^{1/3}}$

Note: for the calculation RR is converted in second (RR (ms)/1,000).

7.6 Vital signs and Physical examination

Vitals signs include body temperature, weight, pulse rate and blood pressure. Blood pressure and pulse rate will be measured after the patient has rested for 3 min. The physical examination include general appearance, skin, head and neck, eyes, ears, nose, throat, lymph node palpation, lungs, heart, chest, abdomen, neurological function.

7.7 PK sampling

About two (2) mL of venous whole blood samples will be collected from all of the 1,000 subset patients (using direct venipuncture) into 5 mL tubes containing lithium heparin **or EDTA depending on site specific procedures. This will be done at every PK time point**

making up for a total amount of 8mL to be taken from each participant in this subgroup over the entire study period.

Each sample will **be** promptly centrifuged and then plasma will be stored at approximately – 75 ± 6°C until shipment to the analytical facilities. All details regarding blood and plasma preparations will be provided in a separate manual of operations.

Concentrations of PQ will be measured in plasma samples. Full details of the methodology will be presented in the bioanalytical protocols.

8. SUBJECT SAFETY

8.1 Training

Before the start of the study, the following training programmes will be completed:

The health facility study staff will be trained on the technique of thick blood smear preparation and staining according to the local Standard Operating procedure and on the recording of ECG data.

The medical staff of the HDSS centres as well as the CHA and the health care provider (HCP) will be trained on the tolerability profile of Eurartesim®, on pharmacovigilance procedures (adverse events recording, adverse events management...) and on study specific other procedures (registry maintenance, non-directional/open patient questioning, filling data sheets) and tools.

The referral hospital staff will be trained on how to recognize and record data of patients who have been referred to them who are taking part in this study. The medical staff of the referral hospitals will be trained on the tolerability profile of Eurartesim®, on pharmacovigilance procedures (adverse events recording, adverse events management...) and on study specific other procedures (registry maintenance, non-directional/open patient questioning, filling data sheets) and tools.

8.2 Monitoring of adverse events

Patient charts (completely filled in with the contact visit at Day 5 (±2 days) or visit Day 7 in the subset of 1,000 patients, and with any AE recording reported in the 28 days after the start of the treatment) will be transferred in triplicate to the Principal Investigator (PI) of the HDSS, every six weeks. Chart review meetings shall be organised regularly, during which the PI, his/her team and the people responsible for the pharmacovigilance in the country participating in the study shall review AEs data (intensity, causality, date of event manifestation in relation to the start of treatment and the initial parasite species identified on the patient). They will determine the causal relationship between any of the recorded adverse event and the medicinal product. This relationship shall be recorded on the patient chart. The notification of the occurrence of serious adverse events, severe adverse events and adverse

events of special interest (which follow a specific process upstream), shall be transmitted to the Local Pharmacovigilance Monitoring Committee every three months for identification of a possible signal.

8.3 Definition of Adverse Event and Serious Adverse Event

An **Adverse Event** (AE) is a sign, symptom, syndrome, disease or biological anomaly suffered by a patient or a subject participating in a clinical study and receiving a medicinal product. This term does not imply a causal relationship with the concerned treatment. Clinical signs typical of an acute malaria episode will not be considered AEs unless the healthcare personnel considers these events as exceptional due to their evolution, their seriousness, or another factor related to these events.

A **Serious Adverse Event** (SAE) is an adverse event which:

- causes death or
- is life-threatening or
- necessitates hospitalisation or prolongs hospitalisation or
- results in invalidity or long-term or significant disability or
- is a congenital defect or malformation or
- is another medically important event.

A decision on medical and scientific grounds is required to assess whether an immediate notification of an event is warranted in other situations, such as medically important events which are not life-threatening, fatal or cause hospitalization, but could endanger the patient or could rendered necessary an intervention to prevent one of the above conditions to develop.

Remark: Examples of such events are intensive care in the emergency room or at home to treat a bronchospasm; a haematological dyscrasia; convulsions or an asymptomatic increase ALT ($\geq$ 10 x ULN) not causing hospitalisation, or the development of drug addiction or abuse.

8.4 Obligation of AE notification

Adverse Events

The AEs, regardless of their seriousness and causal relationship to the study drug, arising between the signature of the informed consent form and the last study visit (as per the protocol), must all be recorded on the patient chart (AE recording section). When possible, the symptoms must be regrouped within a single syndrome or diagnosis. The healthcare personnel shall have to specify the date of manifestation of the event, its intensity, final evolution, the measures taken and the treatment undertaken (if any).

Serious Adverse Events

In case of SAEs, the healthcare personnel must immediately contact the site PI / Investigator for validation of the seriousness and determination of the causality. Subsequently, the procedure described below must be followed:

• SEND (within 24 hours and fax or pdf scanned documents) the signed and dated copy of the "**Serious** Adverse Event form" and the form "SAE complementary information" to INDEPTH-Network secretariat (eurartesim@indepth-network.org), Study Monitor (magyemang-boakye@margancro.org) and the Corporate Safety dept, Sigma-Tau via fax (+39 06 91394007) or e-mail (pharmacovigilance@sigma-tau.it).

• CALL immediately (the same day) the physician of the Sponsor (+254 733 812 613) responsible for safety in case of death or life-threatening events.

• CALL the local responsible person for pharmacovigilance of the project.

• The follow-up of each fatal or life-threatening AE must be provided to the Physician of the Sponsor (INDEPTH-Network), the local person responsible for the pharmacovigilance of the project and the Corporate Safety dept – Sigma-Tau within the same timeline as the initial report (within 24 hours and preferably by e-mail/fax).

• ATTACH to the chart the photocopy of all available results and examinations which were undertaken (and their date). Analysis results must be accompanied by the laboratory normal ranges. Special consideration shall be taken to ensure patient anonymity, and to the correct completion of the patient's study specific identifier in the copies of the source documents provided to the sponsor.

Follow-up of SAE

The healthcare personnel must take all appropriate measures to protect the safety of the patients. Personnel must ensure to document follow-up of the evolution of each adverse event (clinical, biological or other) until resolution or until the stabilization of the patient's status.

All new relevant information concerning the initial SAE shall be recorded on a form "SAE follow-up form" by the nursing staff of the health centre, and shall be validated by the PI / co-PI who shall transfer the form to the to the Physician of the Sponsor (INDEPTH-Network), **the study monitor,** the local person responsible for the pharmacovigilance of the project and the Corporate Safety department – Sigma-Tau.

In case of a serious adverse event, the patient must be followed until complete healing and normalization of all analysis results, or until chronicity of the patient's status. This can imply that the follow-up of the patient must continue beyond the period of follow-up per protocol, and that additional investigations could be requested by the sponsor.

8.5 Adverse event of special interest

An adverse event of special interest (AESI) is an adverse event for which on-going monitoring is appropriate within the context of the study. These events necessitate complementary examinations in order to characterize and understand them.

AESIs in this study can be related to:

- Cardio-toxicity i.e. prolonged QT
- Neurotoxicity/seizures

- Cutaneous reactions/phototoxicity

The study team, as well as the relevant referral facilities, should be trained to take particular notice of symptoms/signs suggestive of the AESIs in this study:

-clinical signs of possible cardio-toxicity/prolonged QT such as:

- Palpitations
- Seizures
- Pounding/pain in the chest area
- Fainting/Syncope.

- clinical signs of possible neurotoxicity/seizures such as:

- Seizures
- Dizziness
- Pins and needles sensations
- Visions disturbance
- Difficulties coordination
- Tinnitus

-clinical signs of possible cutaneous reactions/phototoxicity such as:

- Urticaria
- angioedema
- Skin lesions
- Itching pruritus
- Discoloration
- Dermatitis

In all these cases, patients shall be directed to the health facility for evaluation and recording of all relevant information. If an AESI is confirmed, the PI / investigators should be contacted.

In case of an AESI confirmed by the PI / investigators, the sponsor and the Corporate Safety department of Sigma-Tau shall be informed within 24 hours, even if the event does not satisfy any condition of seriousness**. In this last case, n**otification will occur through the use of an ad hoc AESI form.

In the case of clinical signs of possible cardio-toxicity, an ECG will be performed as soon as the patient arrives at the health facility to look for the following modifications;

- QTcF >500ms
- Increase of QTcF from baseline >60 ms
- T wave liability, or T wave morphologic changes during therapy

The patient will be asked about the time elapsed from Eurartesim® administration and meal.

The study team should be aware of the fact that, although drug-induced prolongation of the QTc interval is usually asymptomatic, signs of the following clinical events could be potentially associated with a QTc prolongation:

- Torsade de pointes
- Sudden death
- Ventricular tachycardia
- Ventricular fibrillation and flutter
- Syncope
- Seizure.

8.6 Pregnancy

In case of pregnancy, the anti-malarial treatment shall be the one recommended by the NMCP. The patient will not be included in the study.

Female patients will be encouraged to communicate to the CHA if they will be pregnant within a period of two months after the start of the Eurartesim® treatment.

The evolution of the pregnancy will be monitored with visits at 3, 6, 9 months and after the delivery. Information on the drugs taken during the pregnancy as well as AEs/SAEs and the health status of the newborn/s will be collected; thereafter the newborn/s will be followed at 6 and 14 weeks.

8.7 Sponsor obligations

Throughout the study, the sponsor shall report expeditiously all unexpected SAEs which are reasonably related to the study drug to the local responsible person for pharmacovigilance, to the Ethics Committee in accordance with the procedure, and to the investigators. The pharmacovigilance person will submit the reported events to the competent authorities in accordance with local regulations.

The sponsor can also report expeditiously all expected SAEs which are reasonably related to the study drug, to the local responsible person for pharmacovigilance. The latter will submit the reported events to the competent authorities in accordance with local regulations.
Each AE not listed as being an expected event in the SmPC and in this protocol shall be considered to be unexpected.

9. STATISTICAL METHODS

The statistical analyses will be performed by INESS using the software package STATA®.

A detailed statistical analysis plan will be generated within three months of study beginning. This plan may be revised during the course of the study in order to take into account the protocol amendments, if any, and to address unexpected problems encountered during the conduct of the study, which could affect the planned analyses. A final statistical analysis plan shall be produced before the database lock.

9.1 Analysis of data at study entry

A descriptive analysis of all data recorded at study entry will be carried-out in order to characterize the population under study.

9.2 Analysis in the whole sample

Analysis in the whole sample and in the Subset population

The statistical analysis will be mainly descriptive. The estimates of the incidence of adverse events will be based on crude rates. All estimates will be complemented with appropriate 95% confidence intervals.

The adverse events shall be regularly coded using MedDRA before database lock.

All SAEs and AESIs will be reviewed centrally by an independent safety monitoring panel.

Incidences will be computed on the following classes of AEs, overall and by SOC/PT:

- All AEs
- Serious AEs
- Serious AEs related to study treatment
- AEs of special interest (AESIs)
- AEs which caused early discontinuation of Eurartesim®
- AEs related to Eurartesim® treatment

Specific analyses will be carried-out on the AESIs. Correlation analyses will be carried-out in order to verify if their occurrence is more frequent with the previous/concomitant use of other drugs (specific classes to be determined in the statistical analysis plan), including antimalarials, in presence of co-morbidity (specific classes to be determined in the statistical analysis plan), or in presence of parasitaemia.

Compliance will be computed in the whole population and in the subset of **about 1,000** patients, calculating for each patient the ratio between the number of tablets taken and the number of tablets that the patient should have taken. Two groups of patients shall be described: compliant = 100% treatment compliance and non compliant = other cases. The safety profile of compliant vs non-compliant patients will be compared.

Additional analysis in the sub-set population

QTc data will be recorded and centrally analysed according to the method described in the ICH guideline E14. The impact of all recorded covariates on QTc prolongation will be investigated through analysis of covariance techniques.

For the AESIs possibly related to cardio-toxicity, a correlation analysis with QTc values recorded at the visit of Day 3 (both before and 3-4 hour after treatment administration) will be carried-out. Occurrence of these events will also be studied with respect to distance from food intake.

Laboratory values will be analysed after normalization. Shift tables from normal to abnormal and vice-versa will be generated.

All the analyses previously described will be also performed by class of age and by type of Plasmodium.

9.3 Determination of sample size

According to the cases registered in the previous years in the area where the study is performed, it should be possible to include approximately 10,000 cases of malaria confirmed by a parasitological diagnosis or diagnosed according to WHO criteria. This number of cases will allow identification of "rare" adverse events (incidence: 1 case/3,000 patients) with a 95% probability.

The sample size computation for the nested study (~1,000 patients) has been also based on feasibility considerations. The focus of this sub-set study will be on the combination of AESIs related to cardio-toxicity/prolonged QT (see above). In this sub-set, there will be a probability of 0.95 (0.865) of observing at least one cardiac AESI assuming that the true incidence of this event is 0.003 (0.002). For such a sample size, if no events have been observed, the upper limit of the 95% confidence interval for the probability of such event is 0.003.

10. DATA MANAGEMENT

10.1 Collection and validation of data

At the inclusion of a patient in the study, the data will be collected on **study specific logs and CRFs** . These forms will report the initials of the patient's name/middle/surname, the patient ID number and a code shall allow the identification of the people (HF Study team and CHA) who have recorded the information in the patient chart.

At least twice a week, patient charts will be provided by HF study team to the CHA of the concerned villages (depending on the available human resources in the designated study health; conversely, CHA can regularly pick up the charts in the health facilities) in order to allow the CHA to perform the patient's follow-up contact at Day 5 (±2 days).

As per the agreed monitoring schedule, the study monitor shall perform additional monitoring visit as necessary to verify completeness of such forms, to verify the registries of the patients with at least 28 days of follow-up from the enclosure in the study.

There will be 100% source data verification of all informed consent forms, AE and SAE recordings by the study monitor during monitoring visits of the 1,000 cohort and 5% of the 9,000 cohort and if there is more than 10% errors in the 5% then 100% review will be done.

Patient's forms will be transferred to the site Principal Investigator (PI) or designee at the HDSS. Form review meetings will be organised regularly, during which the PI, his/her team and the people responsible for the pharmacovigilance in the countries participating in the study shall determine the causal relationship between any of the recorded adverse event and the investigational product. This relationship shall be recorded on the patient form. These forms will be also reviewed by the study team to verify their completeness. One of the three copies of these charts shall subsequently be transmitted for data-entry and then entered into the local database, while the original chart shall be sent to the sponsor and the third copy of the chart shall be kept by the responsible persons for pharmacovigilance in the countries participating in the study.

Once the data is entered, verified and cleaned it will be uploaded into the central database based at Accra.
The sponsor study coordinator shall organise in a regular fashion the capture of these data by each HDSS together with the results of the thick blood smears of those patients.
Queries on the entered data will be generated by the central data manager based in Accra and periodically sent to the HDSS centres for resolution. The concerned HF and HDSS team shall have to answer by confirming or amending the initial data on the specific query form. A copy of the answered query form will be attached to the local copies of the patient chart. The returned answered query will be attached at the original copy of the patient chart stored by the sponsor.

After data capture, the adverse events shall be classified by MedDRA system organ class, preferred term.

The central database and original patient charts will be stored by the Sponsor in Accra, Ghana.

10.2 Quality control of data on site

Approximately 10% of the forms collected in each health facility shall be verified against the centre's registries and charts.

In case specific problems are encountered in a centre, the percentage of charts verified shall be increased and corrective actions shall be implemented.

The quality control shall be undertaken by a qualified monitor, assigned by the sponsor.

10.3 Quality control of the preparation of thick blood smear slides

The sample preparation technique will be quality controlled by one of the trainers. This person shall visit each site during the course of the study to verify the microscopic slides.

These visits shall be frequent at the start of the study, then may become decreasingly frequent if the quality of the samples is satisfactory. In case anomalies are detected during these study visits, or during the examination of the samples, the concerned Health Agent shall receive additional training.

A provisional schedule or calendar of supervision visits shall be initiated at the end of the training phase.

10.4 Quality control of thick blood smear sample reading

10% of the microscopic slides shall be quality controlled by a second qualified microscopist, who is independent from the centre where the initial microscopy took place. The procedures for quality control will be detailed in an ad hoc specification protocol.

10.5 Data-entry

The data entry will be performed at the HDSS centres by trained teams. The sponsor will develop the source documents and CRF templates and database that will be used for data capture and data entry. The source documents will be completed by the study teams at the health facilities where the patients are seen and followed up. A schedule will be developed at every centre for the transmission of completed forms to the HDSS PI. The completeness of **all** forms will be verified at the health facilities by internal quality control checks before being logged out and sent to the HDSS. Upon receipt at the HDSS **all** forms will be logged in and verified for completeness, consistency and legibility. The forms will then be accepted and handed over to the data entry personnel for data entry.

In case of need, queries will be sent **via email** to the health facility **and HDSS** teams for resolution of the questions and then returned back to the data entry centre. The data will be entered, verified and cleaned. All the data will be backed up and archived daily. A 1**0**0 % QC check of the data base with the forms will be performed **by site study team.**
Once the data set is verified by the data manager and deemed cleaned it will be archived and transferred monthly to the central database in Accra by the data manager.

10.6 Data cleaning and data base locking

Monthly data base locks will be implemented across all HDSS. Data contained in the monthly locked database will be transferred to the central database in Accra and no alterations of such data can be performed afterwards by the HDSS. At the end of the study the centrally aggregated data, will be verified by the sponsor team in collaboration with Sigma-Tau. Once declared clean, the database will be locked before data analysis is performed.
A copy of the data base will be provided to Sigma-Tau for the additional Regulatory analysis.

11. TASKS AND RESPONSIBILITIES

11.1 Local Pharmacovigilance Monitoring Committee

This committee shall be in charge of the regular review (not more than every four months) of the listings of the adverse events collected during the study. The committee shall also receive the notification of serious adverse events, adverse events classified as severe and adverse events of special interest. Each site will have its committee.

In case a signal is identified by the committee, the independent Scientific Review panel shall be contacted as soon as possible.

In a more general manner, all decisions taken by this committee shall be documented in writing and shall be transmitted to the Sponsor for the project located in Accra and attached to the regular report.

The Local pharmacovigilance committee shall be composed of:

- the PI or a designee;
- a member of the District Health Services;
- a member of the National Malarial Control Program (if this is possible in the context of conflicts of Interest);
- a member of the local regulatory authority of the countries participating in the study;

11.2. INESS Safety Monitoring Panel

This panel shall be in charge of the regular review (not more than every four months) of the listings of the adverse events collected during the study. The panel shall also receive the notification of serious adverse events, adverse events classified as severe and adverse events of special interest.

In case a signal is identified by the panel, the Scientific Review Panel shall be contacted as soon as possible.

In a more general manner, all decisions taken by this panel shall be documented in writing and shall be transmitted to the Sponsor for the project located in Accra and attached to the final study report.

The constitution of this panel is a per the existing INESS panel.

MMV and Sigma Tau will be co-opted as non-voting members to the panel.

11.3. Responsibilities of the Scientific Review Panel

This scientific board is composed of scientific experts in the field of pharmacovigilance and/or tropical medicine and of one statistician. If required, it shall be possible to consult *ad hoc* experts in other fields (hepatology, haematology, cardiology, dermatology etc), who can join committee meetings.

The Panel shall be involved in the approval of this protocol and its amendments, and be regularly updated on study progress, in particular on the number of inclusions. The Panel shall receive reports issued by the INESS Safety Monitoring Panel, as well as notifications of serious adverse events and can, if clear signals of safety concern are present, recommend to the study sponsor to halt the inclusion of patients. The decision to halt the study shall be made after discussion and agreement between the members of the Panel, the Sponsor, Sigma-Tau and MMV.

The Panel shall meet for the first time for review of the protocol prior to project initiation. Afterwards they will have meetings or TCs, approximately every six months during the course of the study for review of adverse events, possible confirmation of a signal, and discussion recommendation on the progress of the study. Finally, the board shall meet for the release of the final results.

In between, conference calls can be organized at the request of the sponsor, of an investigator, of a member of the pharmacovigilance committee or of a member of the supervisory committee.

All the decisions taken by the Panel shall be documented in writing; they will be transmitted to the Sponsor for the project located in Accra and attached to the final study report.

11.4. Responsibilities of the clinical staff of the health facilities

The staff shall be responsible to performing the study in accordance with this protocol and in accordance with the legislation and international guidelines under the direction of the local PI.

They are responsible for obtaining an informed consent prior to inclusion in the study, for completing the study documents (registry and patient charts) and for recording all relevant data in relation to the study. Each staff shall ensure that the information reported in the document is precise and accurate.

They must inform the patient of all relevant aspects of the study, including the information in the patient information sheet. All this information shall be provided to the patient in layman's terms.

Prior to study inclusion, the informed consent form shall have to be personally completed (first name, surname), dated and signed by the patient, the patient's parent(s) or a guardian authorised representative. The person who has conveyed the information on the study to the patient shall also sign and date the informed consent form approved by the Ethics Committee. The informed consent forms will be translated into local languages for the benefit of those who do not understand English or French or Portuguese.
In case of patients unable to read and sign the patient information sheet and informed consent form, these documents will be read and explained to the patient in local language in the presence of a witness. The patient or the parent(s)/guardian in case of children below 18 years

old, shall put her/his fingerprint on the informed consent form and the witness shall also sign the consent form to confirm that the patient has consented willingly.

A copy of the information sheet and the signed consent form shall be handed over to the patient or the parent/guardian.

11.5. Responsibilities of the Sponsor

The study sponsor is responsible toward study teams at the HDSS and the health authorities and shall take all reasonable measures to ensure the good conduct of the study with regards to ethics, protocol compliance, integrity and validity of the information recorded in the patient chart and registry as well as with regards to the availability of the adequate resources to ensure a perfect conduct of the study. In this respect, the principal function of the monitoring team is to help the investigator and the sponsor to maintain a high level of ethical, scientific, technical and regulatory standards for all study-related aspects of ethics, regulations and administrative rules.

11.6. Ethical Standards

The study shall be conducted in compliance with the text of the Declaration of Helsinki adopted by the 18th World Medical Association Assembly in 1964, and with its amendments. The study will seek approval from the local IRBs/**ECs.**

11.7. Regulations

This study shall be conducted in accordance with the principles of the Good Clinical Practices (US, (4) & European, (5)).

The study shall be conducted in compliance with the international and national laws and regulations in effect, and in accordance with the applicable directives in Ghana, Tanzania, Burkina Faso and Mozambique in particular concerning the submission to the Ethics Committee and the protection of personal data.

11.8. Data protection

The personal data of the patients which could be included in a sponsor database shall be treated in accordance with all local laws and regulations.

At the time of archiving or management of the personal data pertaining to the nursing staff and / or patients, the sponsor shall take all appropriate measures to secure and protect these data against access by a third non-authorised person.

11.9. Insurance

The Sponsor certifies to have subscribed for this study under its sponsorship, an insurance covering the responsibility of the investigator and his team, which is in agreement with the local laws and recommendations. The sponsor's insurance shall not dismiss the investigator

and his collaborators of their obligation to have their own civil liability insurance in line with the laws in force.

A copy of the insurance certificate shall be available for provision to investigators and / or ethics committees who would request it.

11.10. Confidentiality agreement

All information declared or provided by the sponsor (or a company acting on his behalf) or produced during the study, but not limited to the protocol, observation records and data originating from the trial is confidential. The investigator and / or all other persons working under his responsibility commit to maintain confidentiality and to renounce from disclosing information to a third party without the prior written consent of the Sponsor.

However, this protocol and all other documentation required for the Ethics Committee or the health authorities can be handed over to their representatives, who are subjected to the same confidentiality obligations, which are inherent to their function.

11.11. Record keeping

The investigator and the concerned personnel of the health centres and HDSS must keep all study documentation confidential and must take all necessary measures to prevent accidental or premature destruction of these documents.

The regulations or national laws in force on patient record keeping shall be applied.

11.12. Premature termination of the study

The Sponsor can decide at any time and for whatever reason to prematurely suspend or interrupt the study. The decision and the justification shall be communicated in writing to the Pharmacovigilance Monitoring Committee and the Scientific Review Panel.

The local authorities, EC and competent authorities shall have to be informed in line with local legislation.

11.13. Competent authority inspections

The INESS director and the principal investigators accept to grant direct access to study source dossiers to auditors / inspectors for review, with the understanding that these people are bound by professional secrecy and shall not disclose any identity or medical information of a personal nature.

They shall undertake any effort in support of the audits and inspections by facilitating the access to equipment, data and necessary documents.

The confidentiality of the verified data and the protection of the patients shall be respected during these inspections.

All results and all information resulting from these inspections by the regulatory authorities shall be immediately communicated to the Sponsor.

The INESS director and the principal investigators shall take the appropriate measures in order to lead the corrective actions to all problems identified during the audits or inspections.

12. PROTOCOL AMENDMENTS

Each change to the protocol will be reported in a written amendment which will be signed by both the Principal Investigators and the Sponsor. The signed amendment will be added to the protocol.

Following the national legislation, the protocol amendment may require a regulatory submission (for example to the Ethics Committee) before implementation. Sometimes, an amendment may result in changes to the informed consent form. The Sponsor/Principal Investigators must receive an approval/favourable opinion from the Ethics Committee on the received informed consent form before use.

13. DOCUMENTATION AND UTILISATION OF STUDY RESULTS

13.3. Properties and use of the study data and results

All results, data, documents and inventions obtained, directly or indirectly, from the trial, will be owned by the Sponsor unless a law or local regulation states otherwise. The Sponsor can use or exploit all results for their own use without any limitation of it's industrial property (territory, area, duration) in consultation with the HDSS. A specifically appointed scientific committee will have full access to the final database allowing analysis and universitary communications of the survey results. Sigma-Tau is entitled to obtain copy of the data base to be utilized for producing a safety report to be submitted to the European Medicine Agency and has the right to participate with the Sponsor in the publication of such results.

13.4. Publications

The study has already been registered on www.clinical trial.gov
The scientific committee is responsible for the presentations and/or publications of the results. The results of the study will be submitted to the Committee before each publication. Each subsequent presentation or publication should be approved by the scientific board.

The final decision on the publication of a manuscript/summary/presentation will be taken by the scientific board after notification of the Sponsor in order to allow for an internal review and the possibility of providing comments. Each manuscript, summary, presentation will be submitted to the Sponsor, Sigma-Tau and MMV for internal review and possible comments at least 45 days before the submission to the journal and at least 20 days before the submssion of

the summary. The Sponsor may request that their name and/or the name of one of their employees is present or not present on the publication. The Sponsor may delay each publication or communication during a limited time frame in order to protect the confidentiality or the proprietary information present in the document.

14. REFERENCES

1. WHO World Malaria Report [http://www.who.int/malaria/world_malaria_report_2010/worldmalariareport2010.pdf /wmr2010)
2. Bassat Q, Mulenga M, Tinto H, Piola P, Borrmann S, Menéndez C, Nambozi M, Valéa I, Nabasumba C, Sasi P, Bacchieri A, Corsi M, Ubben D, Talisuna A, D'Alessandro U. Dihydroartemisinin-piperaquine and artemether-lumefantrine for treating uncomplicated malaria in African children: a randomised, non-inferiority trial. PLoS One. 2009 Nov 17;4(11):e7871
3. Valecha N, Phyo AP, Mayxay M, Newton PN, Krudsood S, Keomany S et al. An open-label, randomised study of dihydroartemisinin-piperaquine versus artesunate-mefloquine for falciparum malaria in Asia. PLoS One. 2010 Jul 30;5(7):e11880
4. Owusu-Agyei, S., K.P. Asante, M. Adjuik, G. Adjei, E. Awini, M. Adams, S. Newton, D. Dosoo, D. Dery, A. Agyeman-Budu, J. Gyapong, B. Greenwood and D. Chandramohan, Epidemiology of malaria in the forest-savanna transitional zone of Ghana. Malar J, 2009. **8**: p. 220.
5. Dery, D.B., C. Brown, K.P. Asante, M. Adams, D. Dosoo, S. Amenga-Etego, M. Wilson, D. Chandramohan, B. Greenwood and S. Owusu-Agyei, Patterns and seasonality of malaria transmission in the forest-savannah transitional zones of Ghana. Malar J, 2010. **9**: p. 314.
6. Asante, K.P., L. Abokyi, C. Zandoh, R. Owusu, E. Awini, A. Sulemana, S. Amenga-Etego, R. Adda, O. Boahen, S. Segbaya, E. Mahama, C. Bart-Plange, D. Chandramohan and S. Owusu-Agyei, Community perceptions of malaria and malaria treatment behaviour in a rural district of Ghana: implications for artemisinin combination therapy. BMC Public Health, 2010. **10**: p. 409.
7. Binka FN, Morris SS, Ross DA, Arthur P & Aryeetey ME (1994) Patterns of malaria morbidity and mortality in children in northern Ghana. Transactions of the Royal Society of Tropical Medicine and Hygiene 88, 381-385
8. Koram KA, Owusu-Agyei S, Fryauff DJ, Anto F, Atuguba F, Hodgson A, Hoffman SL, Nkrumah FK. (2003). Seasonal profiles of malaria infection, anaemia, and bednet use among age groups and communities in northern Ghana. Tropical Medicine and International Health 8, 793-802.
9. Appawu M, Owusu-Agyei S, Dadzie S, Asoala V, Anto F, Koram K, Rogers W, Nkrumah F, Hoffman SL, Fryauff DJ. (2004) Malaria transmission dynamics at a site in northern Ghana proposed for testing malaria vaccines. Tropical Medicine and International Health 9, 164-170.
10. Baird JK, Owusu Agyei S, Utz GC, Koram K, Barcus MJ, Jones TR, Fryauff DJ, Binka FN, Hoffman SL, Nkrumah FN (2002). Seasonal malaria attack rates in infants and young children in northern Ghana. Am J Trop Med Hyg. 66(3):280-6.
11. **Owusu-Agyei, S., K.P. Asante, M. Adjuik, G. Adjei, E. Awini, M. Adams, S. Newton, D. Dosoo, D. Dery, A. Agyeman-Budu, J. Gyapong, B. Greenwood and D. Chandramohan, Epidemiology of malaria in the forest-savanna transitional zone of Ghana. Malar J, 2009. 8: p. 220.**
12. **Dery, D.B., C. Brown, K.P. Asante, M. Adams, D. Dosoo, S. Amenga-Etego, M. Wilson, D. Chandramohan, B. Greenwood and S. Owusu-Agyei, Patterns and**

seasonality of malaria transmission in the forest-savannah transitional zones of Ghana. Malar J, 2010. 9: p. 314.

13. **Asante, K.P., L. Abokyi, C. Zandoh, R. Owusu, E. Awini, A. Sulemana, S. Amenga-Etego, R. Adda, O. Boahen, S. Segbaya, E. Mahama, C. Bart-Plange, D. Chandramohan and S. Owusu-Agyei, Community perceptions of malaria and malaria treatment behaviour in a rural district of Ghana: implications for artemisinin combination therapy. BMC Public Health, 2010. 10: p. 409.**
14. Khatib, R. A., G. F. Killeen, et al. (2008). "Markets, voucher subsidies and free nets combine to achieve high bed net coverage in rural Tanzania." Malar J **7**: 98.
15. Smithson, P. (2009). Down but not out. The impact of malaria control in Tanzania. I. H. Institute.
16. Sie A, Louis VR, Gbangou A, Muller O, Niamba L, Stieglbauer G, Ye M, Kouyaté B, Sauerborn R, Becher H : The health and demographic surveillance system (HDSS) in Nouna, Burkina Faso, 1993-2007. Global health Action 2010, 3 :5284.
17. Mueller O, Becher H, van Zweeden AB, Ye Y, Diallo DA, Konate AT, Gbangou A, Kouyate B, Garenne M: Effect of zinc supplementation on malaria and other causes of morbidity in west African children: randomised double blind placebo controlled trial. BMJ 2001, 322:1567.
18. Kouyate B, Sie A, Ye M, De Allegri M, Mueller O: The great failure of malaria control in Africa: a district perspective from Burkina Faso. PLoS Med 2007, 4:e127.
19. De Allegri M., Louis V.R., Tiendrebéogo J., Souares A., Yé M., Tozan Y., Jahn A., Mueller O.: Uni- versal Coverage with Malaria Control Interventions: Achievements and Challenges in Rural Burkina Faso, submitted
20. Tipke M, Louis VR, Ye M, De Allegri M, Beiersmann C, Sie A, Mueller O, Jahn A: Access to malaria treatment in young children of rural Burkina Faso. Malar J 2009, 8:266.
21. Nhacolo AQ, Nhalungo DA, Sacoor CN, Aponte JJ, Thompson R, Alonso P. Levels and trends of demographic indices in southern rural Mozambique: evidence from demographic surveillance in Manhiça district. BMC Public Health. 2006 Nov 30;6:291.
22. Alonso PL, Sacarlal J, Aponte JJ, Leach A, Macete E, Milman J, Mandomando I, Spiessens B, Guinovart C, Espasa M, Bassat Q, Aide P, Ofori-Anyinam O, Navia MM, Corachan S, Ceuppens M, Dubois MC, Demoitié MA, Dubovsky F, Menéndez C, Tornieporth N, Ballou WR, Thompson R, Cohen J. Efficacy of the RTS,S/AS02A vaccine against Plasmodium falciparum infection and disease in young African children: randomised controlled trial. Lancet. 2004 Oct 16-22;364(9443):1411-20.

15. ANNEXES

Annex 1: Declaration of Helsinki
Annex 2: Guidance for gradation of clinical symptoms

ANNEX 1

Declaration of Helsinki

WORLD MEDICAL ASSOCIATION DECLARATION OF HELSINKI
Ethical Principles for Medical Research Involving Human Subjects

Adopted by the 18th WMA General Assembly, Helsinki, Finland, June 1964, and amended by the:
29th WMA General Assembly, Tokyo, Japan, October 1975
35th WMA General Assembly, Venice, Italy, October 1983
41st WMA General Assembly, Hong Kong, September 1989
48th WMA General Assembly, Somerset West, Republic of South Africa, October 1996
52nd WMA General Assembly, Edinburgh, Scotland, October 2000
53rd WMA General Assembly, Washington 2002 (Note of Clarification on paragraph 29 added)
55th WMA General Assembly, Tokyo 2004 (Note of Clarification on Paragraph 30 added)
59th WMA General Assembly, Seoul, October 2008

A. INTRODUCTION

1. The World Medical Association (WMA) has developed the Declaration of Helsinki as a statement of ethical principles for medical research involving human subjects, including research on identifiable human material and data.

   The Declaration is intended to be read as a whole and each of its constituent paragraphs should not be applied without consideration of all other relevant paragraphs.

2. Although the Declaration is addressed primarily to physicians, the WMA encourages other participants in medical research involving human subjects to adopt these principles.

3. It is the duty of the physician to promote and safeguard the health of patients, including those who are involved in medical research. The physician's knowledge and conscience are dedicated to the fulfilment of this duty.

4. The Declaration of Geneva of the WMA binds the physician with the words, “The health of my patient will be my first consideration,” and the International Code of Medical Ethics declares that, “A physician shall act in the patient's best interest when providing medical care.”

5. Medical progress is based on research that ultimately must include studies involving human subjects. Populations that are underrepresented in medical research should be provided appropriate access to participation in research.

6. In medical research involving human subjects, the well-being of the individual research subject must take precedence over all other interests.

7. The primary purpose of medical research involving human subjects is to understand the causes, development and effects of diseases and improve preventive, diagnostic and therapeutic interventions (methods, procedures and treatments). Even the best current interventions must be evaluated continually through research for their safety, effectiveness, efficiency, accessibility and quality.

8. In medical practice and in medical research, most interventions involve risks and burdens.

9. Medical research is subject to ethical standards that promote respect for all human subjects and protect their health and rights. Some research populations are particularly vulnerable and need special protection. These include those who cannot give or refuse consent for themselves and those who may be vulnerable to coercion or undue influence.

10. Physicians should consider the ethical, legal and regulatory norms and standards for research involving human subjects in their own countries as well as applicable international norms and standards. No national or international ethical, legal or regulatory requirement should reduce or eliminate any of the protections for research subjects set forth in this Declaration.

B. PRINCIPLES FOR ALL MEDICAL RESEARCH

11. It is the duty of physicians who participate in medical research to protect the life, health, dignity, integrity, right to self-determination, privacy, and confidentiality of personal information of research subjects.

12. Medical research involving human subjects must conform to generally accepted scientific principles, be based on a thorough knowledge of the scientific literature, other relevant sources of information, and adequate laboratory and, as appropriate, animal experimentation. The welfare of animals used for research must be respected.

13. Appropriate caution must be exercised in the conduct of medical research that may harm the environment.

14. The design and performance of each research study involving human subjects must be clearly described in a research protocol. The protocol should contain a statement of the ethical considerations involved and should indicate how the principles in this Declaration have been addressed. The protocol should include information regarding funding, sponsors, institutional affiliations, other potential conflicts of interest, incentives for subjects and provisions for treating and/or compensating subjects who are harmed as a consequence of participation in the research study. The protocol should describe arrangements for post-study access by study subjects to interventions identified as beneficial in the study or access to other appropriate care or benefits.

15. The research protocol must be submitted for consideration, comment, guidance and approval to a research ethics committee before the study begins. This committee must be independent of the researcher, the sponsor and any other undue influence. It must take into consideration the laws and regulations of the country or countries in which the research is to be performed as well as applicable international norms and standards but these must not be allowed to reduce or eliminate any of the protections for research subjects set forth in this Declaration. The committee must have the right to monitor ongoing studies. The researcher must provide monitoring information to the committee, especially information about any serious adverse events. No change to the protocol may be made without consideration and approval by the committee.

16. Medical research involving human subjects must be conducted only by individuals with the appropriate scientific training and qualifications. Research on patients or healthy volunteers requires the supervision of a competent and appropriately qualified physician or other health care professional. The responsibility for the protection of research subjects must always rest with the physician or other health care professional and never the research subjects, even though they have given consent.

17. Medical research involving a disadvantaged or vulnerable population or community is only justified if the research is responsive to the health needs and priorities of this population or community and if there is a reasonable likelihood that this population or community stands to benefit from the results of the research.

18. Every medical research study involving human subjects must be preceded by careful assessment of predictable risks and burdens to the individuals and communities involved in the research in comparison with foreseeable benefits to them and to other individuals or communities affected by the condition under investigation.

19. Every clinical trial must be registered in a publicly accessible database before recruitment of the first subject.

20. Physicians may not participate in a research study involving human subjects unless they are confident that the risks involved have been adequately assessed and can be satisfactorily managed. Physicians must immediately stop a study when the risks are found to outweigh the potential benefits or when there is conclusive proof of positive and beneficial results.

21. Medical research involving human subjects may only be conducted if the importance of the objective outweighs the inherent risks and burdens to the research subjects.

22. Participation by competent individuals as subjects in medical research must be voluntary. Although it may be appropriate to consult family members or community leaders, no competent individual may be enrolled in a research study unless he or she freely agrees.

23. Every precaution must be taken to protect the privacy of research subjects and the confidentiality of their personal information and to minimize the impact of the study on their physical, mental and social integrity.

24. In medical research involving competent human subjects, each potential subject must be adequately informed of the aims, methods, sources of funding, any possible conflicts of interest, institutional affiliations of the researcher, the anticipated benefits and potential risks of the study and the discomfort it may entail, and any other relevant aspects of the study. The potential subject must be informed of the right to refuse to participate in the study or to withdraw consent to participate at any time without reprisal. Special attention should be given to the specific information needs of individual potential subjects as well as to the methods used to deliver the information. After ensuring that the potential subject has understood the information, the physician or another appropriately qualified individual must then seek the potential subject's freely-given informed consent, preferably in writing. If the consent cannot be expressed in writing, the non-written consent must be formally documented and witnessed.

25. For medical research using identifiable human material or data, physicians must normally seek consent for the collection, analysis, storage and/or reuse. There may be situations where consent would be impossible or impractical to obtain for such research or would pose a threat to the validity of the research. In such situations the research may be done only after consideration and approval of a research ethics committee.

26. When seeking informed consent for participation in a research study the physician should be particularly cautious if the potential subject is in a dependent relationship with the physician or may consent under duress. In such situations the informed consent should be sought by an appropriately qualified individual who is completely independent of this relationship.

27. For a potential research subject who is incompetent, the physician must seek informed consent from the legally authorized representative. These individuals must not be included in a research study that has no likelihood of benefit for them unless it is intended to promote the health of the population represented by the potential subject, the research cannot instead be performed with competent persons, and the research entails only minimal risk and minimal burden.

28. When a potential research subject who is deemed incompetent is able to give assent to decisions about participation in research, the physician must seek that assent in addition to the consent of the legally authorized representative. The potential subject's dissent should be respected.

29. Research involving subjects who are physically or mentally incapable of giving consent, for example, unconscious patients, may be done only if the physical or mental condition that prevents giving informed consent is a necessary characteristic of the research population. In such circumstances the physician should seek informed consent from the legally authorized representative. If no such representative is available and if the research cannot be delayed, the study may proceed without informed consent provided

that the specific reasons for involving subjects with a condition that renders them unable to give informed consent have been stated in the research protocol and the study has been approved by a research ethics committee. Consent to remain in the research should be obtained as soon as possible from the subject or a legally authorized representative.

30. Authors, editors and publishers all have ethical obligations with regard to the publication of the results of research. Authors have a duty to make publicly available the results of their research on human subjects and are accountable for the completeness and accuracy of their reports. They should adhere to accepted guidelines for ethical reporting. Negative and inconclusive as well as positive results should be published or otherwise made publicly available. Sources of funding, institutional affiliations and conflicts of interest should be declared in the publication. Reports of research not in accordance with the principles of this Declaration should not be accepted for publication.

C. ADDITIONAL PRINCIPLES FOR MEDICAL RESEARCH COMBINED WITH MEDICAL CARE

31. The physician may combine medical research with medical care only to the extent that the research is justified by its potential preventive, diagnostic or therapeutic value and if the physician has good reason to believe that participation in the research study will not adversely affect the health of the patients who serve as research subjects.

32. The benefits, risks, burdens and effectiveness of a new intervention must be tested against those of the best current proven intervention, except in the following circumstances:
    - The use of placebo, or no treatment, is acceptable in studies where no current proven intervention exists; or
    - Where for compelling and scientifically sound methodological reasons the use of placebo is necessary to determine the efficacy or safety of an intervention and the patients who receive placebo or no treatment will not be subject to any risk of serious or irreversible harm. Extreme care must be taken to avoid abuse of this option.

33. At the conclusion of the study, patients entered into the study are entitled to be informed about the outcome of the study and to share any benefits that result from it, for example, access to interventions identified as beneficial in the study or to other appropriate care or benefits.

34. The physician must fully inform the patient which aspects of the care are related to the research. The refusal of a patient to participate in a study or the patient's decision to withdraw from the study must never interfere with the patient-physician relationship.

35. In the treatment of a patient, where proven interventions do not exist or have been ineffective, the physician, after seeking expert advice, with informed consent from the patient or a legally authorized representative, may use an unproven intervention if in the physician's judgement it offers hope of saving life, re-establishing health or alleviating

suffering. Where possible, this intervention should be made the object of research, designed to evaluate its safety and efficacy. In all cases, new information should be recorded and, where appropriate, made publicly available.

ANNEX 2
Guidance for the evaluation of the intensity of clinical signs

	Grade 1 MILD	Grade 2 MODERATE	Grade 3 SEVERE	Grade 4 LIFE-THREATENING
**Fever in the following 24h**	N/A	Yes	N/A	N/A
**Weakness**	Small decrease in activity, keeps playing	Moderate decrease in activity, has difficulty to play	No activities, stopped playing	Lethargy
**Muscular or articular pain***	Localised pain, weak intensity	Diffuse pain, weak intensity	Real weakness; limited functions	N/A
**Cephalgia***	Weak, not requiring treatment	Fluctuating, moderate, requiring treatment	Severe, responding to an initial narcotic treatment	Refractory, requiring a repeated narcotic treatment
**Anorexia**	Reduced appetite but still eating solid foods	Reduced appetite, avoiding all solid foods	Refusing breast milk, very reduced appetite, taking neither liquids nor solids (< 2 years ≤ 12 h; > 2 years ≤ 24 h)	Refusing breast milk, very reduced appetite, taking neither liquids nor solids (< 2 years ≤ 12 h; > 2 years ≤ 24 h)
**Nausea***	Small discomfort; continues to ingest normally	Moderate discomfort; ingestion significantly reduced; certain activities are limited	Severe discomfort; no significant ingestion, limited activities	Minimal ingestion of liquids
**Vomiting**	Transient vomiting	Moderate or occasional vomiting	Orthostatic hypotension requiring an infusion	Shock for which hospitalisation for infusion is required
**Abdominal pain***	Weak	Moderate, no treatment required	Moderate to severe – treatment required	Severe – hospitalisation for treatment
**Diarrhea**	Transient; 3-4 liquid stools /day	5–7 liquid stools /day	Orthostatic hypotension or >7 liquid stools /day or infusion required	Shock for which hospitalisation for infusion is required
**Cough**	Transient, no treatment required	Continuous, requiring a treatment	Irrepressible	Cyanosis, violent cough, very difficult breathing
**Pruritus**	Pruritus without rash	Pruritus with rash or Pruritus without eruptions disturbing the sleep	Moderate urticaria	Severe urticaria, anaphylaxis, Quincke's Oedema
**Tinnitus***	Weak	Moderate	Severe including hearing loss	N/A
**Behavioural changes**	Minor concentration difficulties; confusion or minor agitation, normal daily activities; no treatment required	Moderate confusion or agitation; daily activities slightly impacted; minimal treatment required	Severe confusion or agitation; requiring assistance with daily activities; treatment required	Toxic psychosis; hospitalisation required
**Flu symptoms**	Minor nasal congestion, minor rhinitis, without cough	Moderate nasal congestion, moderate rhinitis, with cough	N/A (if severe, classify the symptoms individually)	N/A (in life-threatening circumstances, classify the symptoms individually)

*** Applicable only to children ≥ 3 ans. Respond N/A if younger or for those not able to respond.**

Reference – Based upon the WHO toxicity grading scale for determining the severity of adverse events

ANNEX 3 : Guidance for the evaluation of the intensity of clinical signs (continued)

	Grade 1<br>MILD	Grade 2<br>MODERATE	Grade 3<br>SEVERE	Grade 4<br>LIFE-THREATENIING
**Convulsion**	N/A	N/A	Localised or general	Epileptic state
**Temperature* (tympanic)**	38.0-38.4°C	38,5-40.0°C	> 40.0°C	Persistent fever, equal to >40° during more than 5 days
**Dehydration ****	Normal skin to touch, hydrated mucosa, tears are present, normal eyes, flat fontanel, consolable, regular pulse, normal micturition	Dry skin, dry mucosa, sunken eyes, lack of tears, soft fontanel, irritable, slightly accelerated pulse, reduced micturition	Moist and cold skin, dried skin, completely sunken eyes, no tears, sunken fontanel, lethargic, rapid pulse, no micturition	
**Facial oedema**	Present, minor swelling of the eyes	Moderate swelling of the eyes and the face	Severe swelling of the eyes, the face and the mucosa – impossible to open the eyes	The respiratory system is affected
**Icterus**	Mild subconjunctival icterus	Moderate subconjunctival icterus, mucosa moderately yellow	Severe subconjunctival icterus and icterus of the skin	N/A
**Thorax**	Slightly accelerated breathing (with regard to age and temperature), evanescent or localised rhonci	Moderately accelerated breathing, diffuse or persistent rhonchi	Rapid breathing (< 2 months > 60, 2-12 months > 50, 1-5 years > 40, adults > 30)* dilatation and retraction of the nostrils	Cyanosis
**Abdomen**	Normal abdominal sounds, slight local sensibility and/or hepatomegaly exceeding the costal margin with 2-4 cm and/or palpable spleen and/or presence of umbilical hernia	Slightly abnormal abdominal signs or moderate or diffuse sensibility and/or mild or moderate hepatomegaly (exceeding the costal margin with 4-6 cm) and/or grade 4 splenomegaly (palpable spleen until midway between the navel and the public symphysis	Very abnormal abdominal sounds, pain and resistance during palpation and/or hepatomegaly exceeding the costal margin > 6 cm and/or splenomegaly grade 5 (palpable spleen at beyond midway between the navel and the public symphysis)	No abdominal sound. Contracture
**Skin†**	Localised skin eruption, erythema or pruritus	Desquamation, diffuse maculopapular eruption	Vesicles, moist desquamation or ulceration	Exfoliative dermatitis, implication of mucosa or multiform erythema or suspicion of Stevens Johnson or a necrosis requiring a chirurgical intervention

	**Grade 1**<br>**MILD**	**Grade 2**<br>**MODERAT**	**Grade 3**<br>**SEVERE**	**Grade 4**<br>**LIFE-THREATENIING**
**Hearing**	< 4 years : N/A<br>> 4 years: unilateral reduction of the hearing capacity	< 4 years: N/A<br>> 4 years: severe bilateral or unilateral reduction of the hearing capacity	< 4 years: Any reduction of the hearing capacity<br>> 4 ANS : severe bilateral reduction of the hearing capacity	N/A
**Attempt to pick-up tablets**	Difficulty in grasping the tablet though capable	Cannot grasp the tablet without dropping the tablet	Cannot grasp the tablet	N/A
**Other symptoms/ signs (not described elsewhere)**	No treatment; only monitoring	May require a minimal intervention and monitoring	Requires medical care and possible hospitalisation	Requires active medical care or hospitalisation

***Reference – DMID Toxicity Table for Children, May 2001, drug fever (rectal)**
**** Reference - The Harriet Lane Handbook, 15th edition, 2000**
† Reference - WHO toxicity grading scale for determining the severity of adverse events
